# Consensus recommendations on dosing and administration of medical cannabis to treat chronic pain: results of a modified Delphi process

**DOI:** 10.1186/s42238-021-00073-1

**Published:** 2021-07-02

**Authors:** Arun Bhaskar, Alan Bell, Michael Boivin, Wellington Briques, Matthew Brown, Hance Clarke, Claude Cyr, Elon Eisenberg, Ricardo Ferreira de Oliveira Silva, Eva Frohlich, Peter Georgius, Malcolm Hogg, Tina Ingrid Horsted, Caroline A. MacCallum, Kirsten R. Müller-Vahl, Colleen O’Connell, Robert Sealey, Marc Seibolt, Aaron Sihota, Brennan K. Smith, Dustin Sulak, Antonio Vigano, Dwight E. Moulin

**Affiliations:** 1 Pain Management Centre, Imperial College Healthcare NHS Trust, London, UK; 2 Department of Family and Community Medicine, University of Toronto, Toronto, ON Canada; 3 CommPharm Consulting, Barrie, ON Canada; 4 Canopy Growth Corporation, São Paulo, Brazil; 5 Department of Pain Medicine, The Royal Marsden Hospital, London, UK; 6 The Institute of Cancer Research, London, UK; 7 Department of Anesthesia and Pain Medicine, Toronto General Hospital, University Health Network, University of Toronto, Toronto, ON Canada; 8 Department of Family Medicine, McGill University, Montreal, QC Canada; 9 Institute of Pain Medicine, Rambam Health Care Campus, The Technion, Israel Institute of Technology, Haifa, Israel; 10 Vertebralis Spine Center, Rio de Janeiro, Brazil; 11 Department of Anaesthesiology and Pain Management, Helen Joseph Hospital, Johannesburg, South Africa; 12 Pain Rehab, Noosa Heads, Australia; 13 Department of Anaesthesia and Pain Management, The Royal Melbourne Hospital, Melbourne, Australia; 14 Faculty of Medicine, Dentistry and Health Sciences, The University of Melbourne, Melbourne, Australia; 15 Clinic Horsted, Copenhagen, Denmark; 16 Faculty of Medicine, The University of British Columbia, Vancouver, BC Canada; 17 Hannover Medical School, Department of Psychiatry, Social Psychiatry and Psychotherapy, Hannover, Germany; 18 Department of Physical Medicine and Rehabilitation, Stan Cassidy Centre for Rehabilitation, Fredericton, NB Canada; 19 Cannabinoid Medicine Specialist, Victoria, BC Canada; 20 Algesiologikum– Centers for Pain Medicine, Day Clinic for Pain Medicine, Munich, Germany; 21 The University of British Columbia, Faculty of Pharmaceutical Sciences, Vancouver, BC Canada; 22 CTC Communications, Medical Division, Mississauga, ON Canada; 23 Integr8 Health, Falmouth, Maine USA; 24 Department of Oncology, McGill University, Montreal, QC Canada; 25 Departments of Clinical Neurological Sciences and Oncology, Earl Russell Chair of Pain Medicine, Western University, 800 Commissioners Road East, London, ON N6A 5W9 Canada

**Keywords:** Medical cannabis, Chronic pain, Cannabidiol, CBD, Tetrahydrocannabinol, THC, Delphi process

## Abstract

**Background:**

Globally, medical cannabis legalization has increased in recent years and medical cannabis is commonly used to treat chronic pain. However, there are few randomized control trials studying medical cannabis indicating expert guidance on how to dose and administer medical cannabis safely and effectively is needed.

**Methods:**

Using a multistage modified Delphi process, twenty global experts across nine countries developed consensus-based recommendations on how to dose and administer medical cannabis in patients with chronic pain.

**Results:**

There was consensus that medical cannabis may be considered for patients experiencing neuropathic, inflammatory, nociplastic, and mixed pain. Three treatment protocols were developed. A routine protocol where the clinician initiates the patient on a CBD-predominant variety at a dose of 5 mg CBD twice daily and titrates the CBD-predominant dose by 10 mg every 2 to 3 days until the patient reaches their goals, or up to 40 mg/day. At a CBD-predominant dose of 40 mg/day, clinicians may consider adding THC at 2.5 mg and titrate by 2.5 mg every 2 to 7 days until a maximum daily dose of 40 mg/day of THC. A conservative protocol where the clinician initiates the patient on a CBD-predominant variety at a dose of 5 mg once daily and titrates the CBD-predominant dose by 10 mg every 2 to 3 days until the patient reaches their goals, or up to 40 mg/day. At a CBD-predominant dose of 40 mg/day, clinicians may consider adding THC at 1 mg/day and titrate by 1 mg every 7 days until a maximum daily dose of 40 mg/day of THC. A rapid protocol where the clinician initiates the patient on a balanced THC:CBD variety at 2.5–5 mg of each cannabinoid once or twice daily and titrates by 2.5–5 mg of each cannabinoid every 2 to 3 days until the patient reaches his/her goals or to a maximum THC dose of 40 mg/day.

**Conclusions:**

In summary, using a modified Delphi process, expert consensus-based recommendations were developed on how to dose and administer medical cannabis for the treatment of patients with chronic pain.

**Supplementary Information:**

The online version contains supplementary material available at 10.1186/s42238-021-00073-1.

## Background

Cannabis is being legalized and/or decriminalized across the globe and hundreds of thousands of patients are currently being treated with medical cannabis (Abuhasira et al. 2018; Lintzeris et al. 2020). Patient-reported data indicate that chronic pain management is one of the most common reasons for medical cannabis use (Reiman et al. 2017; Boehnke et al. 2019; Kosiba et al. 2019; Azcarate et al. 2020). Chronic pain affects close to 2 billion people worldwide and is associated with impairment in physical and emotional function, reduced participation in social and vocational activities, and lower perceived quality of life (Dueñas et al. 2016; Hylands-White et al. 2017; Vos et al. 2017). In patients with chronic pain, medical cannabis treatment has been associated with an improvement in pain-related outcomes, increased quality of life, improved function, and a reduced requirement for opioid analgesia (Abrams et al. 2011; Haroutounian et al. 2016; National Academies of Sciences 2017; Cooper et al. 2018; Rod 2019; Sagy et al. 2019; Johal et al. 2020; Safakish et al. 2020; Okusanya et al. 2020).

Despite the increased global use of medical cannabis to manage pain, systematic reviews and meta-analyses report low to substantial levels of evidence to support the use of cannabis and cannabinoids for the treatment of chronic pain (Russo 2007; Whiting et al. 2015; Allan et al. 2018; National Academies of Sciences 2017; Stockings et al. 2018; Mücke et al. 2018; Häuser et al. 2018; Johal et al. 2020; Safakish et al. 2020; Okusanya et al. 2020). Explanations as to why some describe the level of evidence is low may include limited availability of investigational products due to legal status, lack of standardization of cannabis products, lack of standardization of product administration, and overemphasis on pain scores to define efficacy. However, despite the low to moderate level of evidence, patients are being treated with medical cannabis across the world.

Therefore, the lack of randomized control trial evidence combined with the practical reality that patients are receiving a pharmaceutically active drug creates an atypical clinical scenario that necessitates expert guidance from experienced clinicians on how to safely and, perhaps, effectively dose and administer medical cannabis.

The recommendations presented herein were developed as practical guidance for clinicians who may have limited experience with prescribing or recommending (if patient is in USA) medical cannabis. It is important to note that every patient is different and medical cannabis treatment, like most other therapies, should be individualized to the patient. Shared treatment decision-making with the patient is important and establishing treatment goals during the initial medical consultation may enhance patient outcomes and adherence to medical cannabis treatment. The intent is to provide clinicians with safe and effective medical cannabis prescribing protocols, which may be considered when a clinician decides to include medical cannabis in a patient’s treatment regimen.

## Methods

**Fig. 1:**
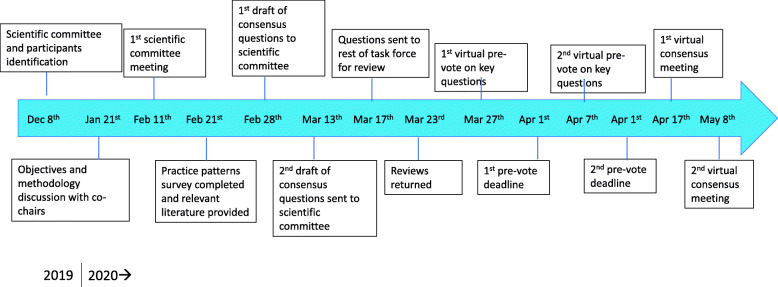
Timeline and Flow of modified Delphi process

To address the unmet need for clinical guidance on the safe and effective use of medical cannabis for chronic pain, and to build on previous recommendations from MacCallum and Russo (2018) and Boehnke and Clauw (2019), we developed a modified Delphi process (Dalkey and Helmer 1963; Dalkey 1969; Saad et al. 2019; Oude Voshaar et al. 2019) to establish expert consensus-based recommendations on the dosage and administration of medical cannabis (Fig. 1). The modified Delphi process has been used extensively in health care settings to provide consensus-based recommendations on important clinical questions where randomized control trial data is lacking (Hasson et al. 2000).

A global task force of twenty individuals was recruited based on extensive clinical experience and/or high academic interest in prescribing and managing patients on medical cannabis for the treatment of chronic pain (Table 1). The panel was selected based on clinical experience prescribing medical cannabis, research with medical cannabis, and a focus on inclusion of representatives from different countries. Upon recruitment, the task force participants completed a practice patterns survey (Additional file 1) to gain insights into how clinicians around the world were treating patients with medical cannabis. After the practice profile was completed, nine recent articles were provided to the task force (Habib and Artul 2018; Banerjee and McCormack 2019; Crawley et al. 2019; Maher et al. 2019; Boyaji et al. 2020; Johal et al. 2020; Montero-Oleas et al. 2020; Wong et al. 2020; Gulbransen et al. 2020). An initial draft of 37 consensus questions was developed based on the practice patterns survey and reviewed for rationale and applicability to clinical practice by a nine-member scientific committee. After review and scientific committee approval, an updated version was distributed to the other task force participants for their review of its rationale and applicability.

**Table 1:** Global task force on medical cannabis dosing and administration for treatment of chronic pain

Last name	First name	Speciality	Country
Bell	Alan	Family Medicine	Canada
Bhaskar	Arun	Pain Medicine	United Kingdom
Brown	Matthew	Pain Medicine	United Kingdom
Clarke	Hance	Anesthesiology	Canada
Cyr	Claude	Family Medicine	Canada
Eisenberg	Elon	Neurology and Pain Medicine	Israel
Ferreira	Ricardo	Pain Medicine	Brazil
Frohlich	Eva	Anesthesiology and Pain Management	South Africa
Georgius	Peter	Pain Medicine	Australia
Hogg	Malcolm	Pain Medicine	Australia
Horsted	Tina	Pain Medicine	Denmark
MacCallum	Caroline	Internal Medicine	Canada
Moulin	Dwight	Pain Medicine	Canada
Müller-Vahl	Kirsten	General Psychiatry, Neurology	Germany
O'Connell	Colleen	Physical Medicine, Rehabilitation	Canada
Sealey	Robert	Family Medicine	Canada
Seibolt	Marc	Anesthesiology, Pain and Addiction Medicine	Germany
Sihota	Aaron	Primary Care Pharmacy	Canada
Sulak	Dustin	Osteopathic Medicine	United States
Vigano	Antonio	Palliative Medicine	Canada

Once the full task force had reviewed all questions and proposed answers, and all comments had been incorporated; the first round of voting took place on 63 questions using an online survey (Qualtrics, Provo, Utah; (Additional file 2) with the following rules in place:

- For multiple choice questions, consensus is found if ≥ 75% of the responses support one answer. For ranking questions, consensus is found if ≥ 75% of the responses are agree/strongly agree or disagree/strongly disagree. This consensus threshold is similar to previous studies using a modified Delphi method (Diamond et al. 2014; Gillessen et al. 2018).
- There was an “abstain” option for all questions.
- For the purposes of this document, medical cannabis refers to CBD and THC extracted from a cannabis plant.
- The dosing and administration protocol was focused on oral preparations (oils and gel capsules) to support harm reduction from smoking and/or e-vaping (Tashkin 2013; Sangmo et al. 2020), and to nullify the risk of e-cigarette or vaping product use-associated lung injury (EVALI) (Layden et al. 2019).
- It was stressed that clinicians would need to customize the recommendations based on availability and regulations in their region of practice.

The first round of voting established consensus on several topics including the rationale for using medical cannabis, the type of pain medical cannabis could be used to treat, age limitations for CBD, when medical cannabis should be avoided, and what the patient goals of using medical cannabis could be. This first round of voting indicated that the task force members were using medical cannabis for similar patient profiles, but dosing and administration protocols were different. The consensus questions were then revised to focus on key remaining elements, and 55 questions were considered for the second round of voting using online surveys (Additional file 3).

Following analysis of the first two rounds of voting, intended live meeting discussion topics were narrowed down. However, due to the COVID-19 pandemic, the live meeting was converted to a virtual format. Over two virtual meetings, 31 questions were voted on through Zoom Meeting polling software (Zoom Video Communications, San Jose, California, Additional files 4 and 5). The key topics for discussion surrounded the dosing and administration procedures across the different medical cannabis treatment protocols. The other two sections for discussion were breakthrough pain and follow-up recommendations. The task force was encouraged to discuss the question before voting to find common ground if possible.

Phrasing of questions was refined over the rounds of review and voting based on task force feedback. At least 16 members of the task force voted at each of the steps. The reader is directed to Additional file 2, 3, 4 and 5 for all voting results.

### Role of funding source

This work was funded by Spectrum Therapeutics. Spectrum Therapeutics is the medical division of Canopy Growth Corporation, which sells both medical and recreational cannabis. The funder influenced the selection of the task force, and all authors declare they have received funding from Spectrum (Additional file 6). However, the funder had no influence on the design and conduct of the voting and discussions; collection, management, analysis, and interpretation of the data; preparation, review, approval of the manuscript; or decision to submit the manuscript for publication. The sponsor was provided the opportunity to review the manuscript for medical and scientific accuracy and did not suggest any changes to the manuscript.

## Results

### Dosing and administration of medical cannabis to treat patients with chronic pain

**Fig. 2:**
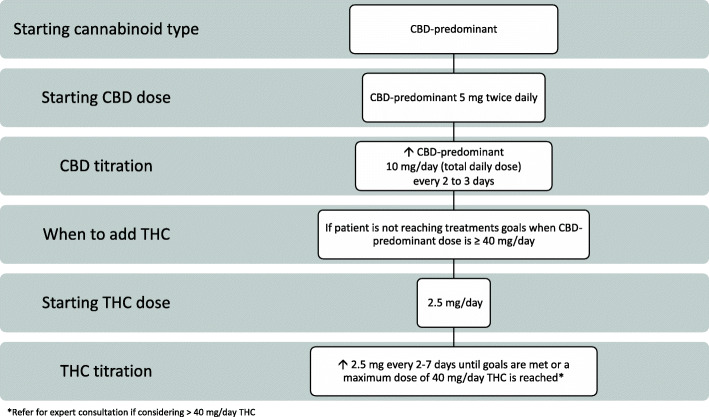
Routine protocol for medical cannabis dosing and administration

**Fig. 3:**
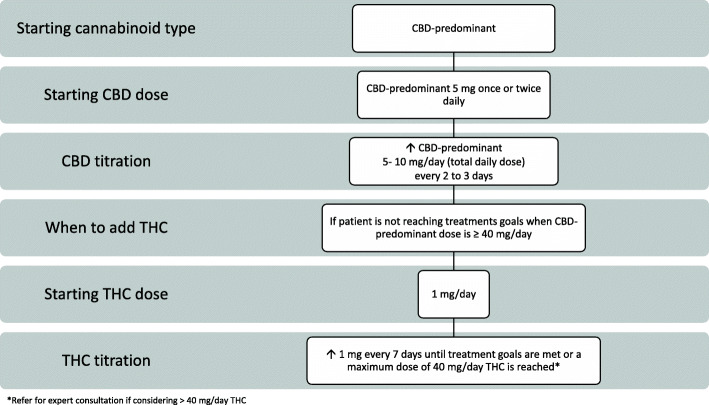
Conservative protocol for medical cannabis dosing and administration

**Fig. 4:**
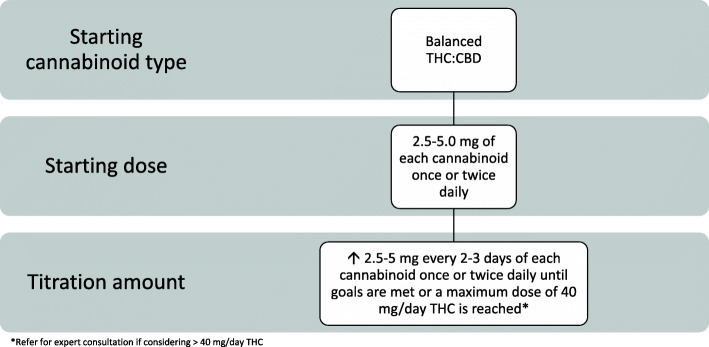
Rapid protocol for medical cannabis dosing and administration

During the Delphi voting, three streams of oral dosing and administration recommendations based on patient need evolved: Routine, Conservative, and Rapid (Figs. 2, 3, and 4). The protocols were developed with a focus on safety and what experienced prescribers observe in their practice to be effective. For each protocol, a starting cannabinoid type was voted on, followed by a titration protocol up to a maximum daily dose recommendation. If necessary, the clinician may consider moving a patient between protocols to individualize the patient’s treatment plan. There was a consensus that medical cannabis may be considered for the treatment of neuropathic pain, inflammatory pain, nociplastic pain, and mixed pain (Sihota et al. 2020). Clinicians should titrate and manage the dosing regimen to reach patient treatment goals, which may be varied and therefore individualized (Table 2).

**Table 2:** Examples of patient treatment goals when using medical cannabis

• Improve quality of life	
• Improve function	
• Improve overall analgesic efficacy	
• Improve self-efficacy	
• Improve sleep	
• Improve mood	
• Reduce anhedonia	
• Reduce anxiety	
• Address breakthrough pain symptoms	
• Address episodic symptoms and exacerbations	
• Improvement in disease specific symptoms and symptom burden	
• Spare opioids and support opioid tapers	
• Reduce benzodiazepine use	
• Reduce skeletal muscle relaxant use	
• Reduce hypnotic use	
• Reduce illicit substance use	
• Reduce nonsteroidal anti-inflammatory drug (NSAID) use	
• Reduce legal substance use (eg, alcohol, tobacco)	
• Mitigate opioid-related adverse effects	
• Reduce opioid withdrawal symptom	

### Routine protocol for medical cannabis dosing and administration

The routine protocol is recommended for most patients (Fig. 2). The Delphi process led to agreement that a patient may initiate with 5 mg twice daily (bid) of a CBD-predominant strain and up-titrate by 10 mg/day (5 mg CBD bid) every 2–3 days up to 40 mg CBD per day. A key reason for choosing to initiate with a CBD-predominant variety was to prioritize safety as CBD is highly tolerable, does not induce euphoria, and has a low risk for adverse effects (Taylor et al. 2018; Larsen and Shahinas 2020). In addition, many CBD-predominant preparations contain a small percentage of THC (Bonn-Miller et al. 2017; Lachenmeier et al. 2020). It was decided that the maximum amount of THC allowed in a CBD-predominant product to be considered for these protocols would be 1:10 THC to CBD. Many global CBD-predominate products contain 0.–2% THC (Bonn-Miller et al. 2017; Corroon et al. 2020; Lachenmeier et al. 2020).

If 40 mg/day CBD-predominant dose does not reach treatment goals, clinicians may consider initiating 2.5 mg of THC per day and titrate by 2.5 mg THC every 2–7 days up to 40 mg/day while maintaining the same CBD-predominant dose. It is recommended to seek expert consultation if considering going above 40 mg/day THC. The THC titration frequency of 2–7 days is a large range to promote tailoring to the patient’s needs.

Clinicians are encouraged to titrate medical cannabis to the effects desired by each patient, as opposed to a specific CBD or THC dose. During the titration phase, the total daily dose of CBD and/or THC can be divided between two to four administrations.

### Conservative protocol for dosing and administration of medical cannabis

The conservative protocol is recommended for patients who may be more sensitive to drug effects (Fig. 3). Clinically frail patients, those with complex comorbidities, polypharmacy, and/or mental health disorders may also be appropriate for the conservative approach. It was agreed a patient may start on a 5 mg once daily dose of a CBD-predominant strain and up-titrate by 5–10 mg every 2–3 days up to 40 mg CBD per day, leveraging twice daily administration when needed. If treatment goals have not been met by 40 mg/day CBD-predominant dose, consider initiating 1 mg of THC and titrating by 1 mg once per week up to 40 mg/day of THC while keeping the same CBD dose. The patient may need a higher THC dose and moving them into the routine stream may be necessary. It is recommended to seek expert consultation if the clinician and patient are considering exceeding 40 mg of THC.

### Rapid protocol for dosing and administration of medical cannabis

The rapid treatment protocol may be considered for patients requiring urgent management of severe pain, palliation, and for those with significant prior use of cannabis (Fig. 4). For patients in palliative care, caution is advised when choosing the medical cannabis protocol as these patients may have higher frailty and a higher risk of terminal delirium, which would make them suitable for the conservative approach as well.

It was agreed that a patient should start on a balanced THC:CBD product of 2.5–5 mg of each cannabinoid once or twice daily and up-titrate every 2–3 days by 2.5–5 mg/day of each cannabinoid until patient goals are met, or to 40 mg THC. If choosing to initiate twice daily with a balanced product, the lower doses would be more appropriate to consider at the beginning. The recommendation to seek expert consultation at 40 mg of THC is also present in the rapid protocol. When considering patients with neuropathic pain, products that contain THC may be more suitable (Andreae et al. 2015; Longo et al. 2020).

### Medical cannabis treatment for breakthrough pain

In patient scenarios where breakthrough pain is common, inhaled medical cannabis can be considered due to the more rapid onset of action and limited duration of action (Huestis 2007). Dried flower vaporization is the preferred mode of administration as opposed to smoking or vaporization of cannabis extracts in an electronic cigarette device (e-vaping), as smoking and e-vaping carry significant health risks. Smoking cannabis is associated with inflammation of the airways and chronic cannabis smokers may experience a heightened risk for bronchitis, respiratory infections, and pneumonia (Tashkin 2013; Volkow et al. 2014; Owen et al. 2014). E-vaping of THC containing products has been associated with a relatively novel but grave lung disease known as e-cigarette or vaping product use-associated lung injury (EVALI) (Layden et al. 2019; King et al. 2020).

When using medical cannabis to manage breakthrough pain, a balanced THC:CBD or THC-predominant product may be used as needed (prn). Clinicians could also consider that breakthrough pain may be suppressed by increasing the dose or frequency of the scheduled oral medical cannabis treatment.

### Follow-up and discontinuation considerations

At the initiation of medical cannabis treatment, clinicians may consider following the patient every 2–4 weeks (Table 3). In individual patients, more frequent follow-up may be needed, particularly at the beginning of the medical cannabis treatment. Once the patient is at a stable dose or sufficiently knowledgeable with medical cannabis dosing and titration, follow-up may occur once every 3 months or even longer thereafter. However, adherence to local jurisdictional guidance may dictate follow-up frequency. The follow-up and discontinuation recommendations were consistent across the three protocols. Discontinuation of medical cannabis treatment should occur if the patient experiences intolerable, moderate, or severe cannabis-related adverse effects, the maximum agreed upon dose is reached and does not benefit the patient, and/or the patient has misuse or diversion associated with cannabis. Reporting of adverse events should be congruent with regional regulatory requirements.

**Table 3:** Follow-up and Monitoring

Scenario	Recommendation
• When initiating medical cannabis for chronic pain, patients should be seen:	• Every 2 to 4 weeks
• When the patient is stabilized on cannabis, the recommended follow-up is:	• Every 3 months although some patients may require more frequent monitoring, or local jurisdictions may legislate monitoring at specific time intervals
• Medical cannabis discontinuation should occur:	• If the patient experiences moderate or severe cannabis-related adverse effects, the maximum agreed upon dose in milligrams is reached and does not benefit the patient, and/or the patient has misuse or diversion associated with cannabis

### Additional safety considerations for medical cannabis use

#### Patients who should avoid medical cannabis

There was consensus that individuals with psychotic disorders, unstable cardiovascular disorders, who are pregnant, who are planning to become pregnant, and/or who are breastfeeding, should avoid medical cannabis, similar to previous guidance documents ([CSL STYLE ERROR: reference with no printed form.]; National Academies of Sciences 2017; Canadian Medical Association 2020). The contraindications associated with medical cannabis are more closely linked to THC, but as discussed, CBD-predominant products may contain THC.

#### Age ranges

There was consensus for no minimum or maximum age limitation for CBD. Although it was agreed no upper age limit for THC use was necessary, there was debate regarding the minimum age recommendation for THC use, but no consensus was found. It has been reported that the human nervous system is not fully developed until 25 years of age, but different jurisdictions around the world have put varying age limits in place (Arain et al. 2013; Casey et al. 2013). In addition, it is unknown whether treatment with medical cannabis supervised by a physician influences brain development in minors. The recommendation for age limits therefore is to follow the local government regulations and consider the clinical risk-benefit ratio to each individual patient.

#### Drug-drug interactions

Drug-drug interactions should be considered (Balachandran et al. 2021). THC is a substrate of CYP3A4 and CYP2C9 while CBD is a substrate of CYP3A4 and CYP2C19 (Antoniou et al. 2020) CBD and THC may also inhibit or stimulate drug transporter P-glycoprotein (Zhu et al. 2006). Direct-acting oral anticoagulants all contain warnings to avoid use with drugs that inhibit CYP3A4 and P-glycoprotein. Caution is strongly encouraged when coadministering medical cannabis with direct-acting anticoagulants ( XARELTO® (rivaroxaban), 2020; https://www.pfizer.ca/sites/default/files/201910/ELIQUIS_PM_229267_07Oct2019_Marketed_E.pdf, 2020; https://www.boehringer-ingelheim.ca/sites/ca/files/documents/pradaxapmen.pdf, 2020), warfarin (Yamreudeewong et al. 2009; Yamaori et al. 2012), drugs metabolized by CYP2C19 (e.g., clopidogrel (Kazui et al. 2010) and clobazam (Geffrey et al. 2015; Cox et al. 2019), checkpoint inhibitors (e.g., PD-1 (Taha et al. 2019), and immunotherapy agents (e.g., tacrolimus (Leino et al. 2019). In addition, awareness around the potential reduced efficacy of theophylline and clozapine is important (Cox et al. 2019).

## Discussion

The modified Delphi process led to the development of three treatment protocols to support dosing and administration of medical cannabis in patients with chronic pain. The clinician may consider moving patients across the streams as a means to tailor the approach. Patient participation in the treatment decisions may enhance adherence and the likelihood of improved patient outcomes. The clinical success of medical cannabis should not be limited to pain scores and should consider improvements in function and quality of life.

### Routine CBD dosing and administration

There was considerable debate around the starting cannabinoid type for routine dosing. It was not until the last round of voting that the group reached consensus to start with a CBD-predominant strain. A deciding factor was ultimately the safety profile of CBD. Purified CBD has been shown to be safe and well tolerated up to 6000 mg (Taylor et al. 2018). CBD at doses ranging from 10 to 20 mg/kg/day is effective as an add-on therapy to reduce refractory seizures in two pediatric populations, Lennox-Gastaut syndrome, and Dravet syndrome (Lattanzi et al. 2018). CBD has also been studied in social anxiety where CBD doses ranging from 25 to 600 mg per day has been shown to be effective, as reviewed in Skelley et al. (2020). Our recommendations are much lower than those used in reducing seizures and are at the lowest end of dosing for social anxiety.

There are some data to suggest that CBD may support pain relief and quality of life. In a recent patient-reported outcomes audit study from New Zealand (*n* = 400), CBD was well-tolerated and improved pain outcomes and quality of life (Gulbransen et al. 2020). The CBD doses used in this study ranged from 40 to 300 mg/day, but there was no statistical association between CBD dose and patient-reported benefit. In a single-arm prospective cohort study investigating the effect of CBD from hemp on opioid use over 8 weeks, CBD reduced opioid use and improved quality of life (Capano et al. 2019). In this study, over 90% of the participants used a dose of 30 mg/day CBD. In a commissioned review by the Australian government, CBD below 60 mg/day was deemed tolerable and safe (Goods Administration 2020). In line with these publications, our Delphi process with global experts in medical cannabis led to the recommendation that in the absence of achieving treatment goals by 40 mg/day of CBD, THC should be considered.

Another deciding factor in choosing CBD-predominant as the initiating product was the fact that many CBD-predominant preparations contain a small percentage of THC (Bonn-Miller et al. 2017; Lachenmeier et al. 2020). If the ratio of THC to CBD is 1:20, a patient taking 40 mg of a CBD-predominant product is also receiving 2 mg of THC. Two milligrams of THC is close to the recommended initiating dose of 2.5 mg. Unexpectedly, experiencing the psychotropic effects of THC may be undesirable for the patient, and treating clinicians should always be aware of the THC concentration within any given product.

Unlike THC, the mechanism of action of CBD is not believed to be primarily through its binding to the cannabinoid receptor. CBD is thought to exert its action on G-coupled protein receptors, transient receptor potential (TRP) channels, reducing intracellular transporters of endocannabinoids, and decreasing metabolism of endocannabinoids through its interaction with the enzyme FAAH and the P450 isoenzyme system (Mlost et al. 2020). CBD has a wide spectrum of biological activity, including antioxidant and anti-inflammatory activity (Atalay et al. 2020). Through these mechanisms of action, CBD is thought to improve symptoms in a variety of chronic pain conditions (Mlost et al. 2020). Preclinical trials have demonstrated a potential anti-nociceptive effect of CBD and when combined with other compounds in several pain-related diseases (Atalay et al. 2020; Mlost et al. 2020).

The 40 mg/day dose of a CBD-predominant strain before adding THC is lower than the CBD doses recommended by Boehske and Clauw (2019). However, the cost of CBD may restrict the use of CBD at high doses (Gulbransen et al. 2020). Moving forward, purified isolates of CBD will likely become more available such that the concern around THC inclusion with CBD-predominant product will be unnecessary.

Sihota et al. recently examined how to use medical cannabis to support opioid tapering (Sihota et al. 2020). The modified Delphi process was also applied in this report to pragmatically align on how to titrate medical cannabis while reducing the opioid dose. This report differs from the present report as we did not specifically consider opioid sparing but considered all patients living with chronic pain. However, similar recommendations on how to dose and administer medical cannabis were observed across the two studies, i.e., start with CBD and titrate THC for most patients. The main difference between the two studies is that the medical cannabis recommendations for opioid tapering are larger in range, while we have provided three titration protocols that may be used depending on the patient. It is encouraging that two Delphi processes resulted in similar recommendations.

### Routine THC dosing and administration

In line with two previous clinical dosing and administration recommendation documents (MacCallum and Russo 2018; Boehnke and Clauw 2019), it was agreed that an initiating THC dose of 2.5 mg was appropriate. A large number of studies in various indications, including chronic pain, have observed that in most patients, the analgesic effects of THC start between 2 and 2.5 mg THC (Beal et al. 1995). It is important to note that analgesic effects of THC in chronic neuropathic pain in humans have been shown to occur at plasma levels well below those associated with euphoria (Ware et al. 2010; Wallace et al. 2020). Therefore, the patient may not need to experience the psychotropic effects of THC to achieve pain relief. However, before considering THC, clinicians should review local jurisdictional regulations on THC, as local guidance on THC may differ from CBD and require additional attention.

There was consensus that the daily dose of THC should not exceed 40 mg unless coupled with expert consultation. As the initiating dose is 2.5 mg, the clinician should titrate slowly with THC and ensure the patient is comfortable with each increasing dose. If considering THC above 40 mg, a consult with a cannabinoid specialist or an experienced medical cannabis clinician is highly recommended as tolerance to cannabis may be developing (Nguyen et al. 2018; Wilkerson et al. 2019).

When considering the pharmacodynamics of orally ingested THC, a recent crossover study examining 17 healthy adults who had not consumed recreational or medical cannabis for at least 60 days, completed four experimental sessions where they ingested 0, 10, 25, or 50 mg of THC (Schlienz et al. 2020). Subjective effects, vital signs, cognitive/psychomotor performance, and blood THC concentrations were assessed before, and then every 30 min for 8 h post ingestion. The 10 mg THC dose produced subjective drug effects and elevated heart rate but did not impact cognitive/psychomotor performance. The 25 and 50 mg doses of THC elicited pronounced subjective effects and impaired cognitive and psychomotor functioning compared to placebo. Subject-reported “good drug effect” was similar between the three doses, but the risk of “bad drug effect” increased with the 25 and 50 mg of THC doses. Although there is wide variation, when considering the majority of patients, 10 mg of THC per day is a typical therapeutic dose. If necessary, the tentative maximum daily dose of 40 mg is still safe but is unlikely to be needed often.

When orally administering THC, the pharmacodynamic effects may begin as early as 30 min and continue to rise between 1 and 3 h post ingestion (Grotenhermen 2003; Schlienz et al. 2020). This coincides with whole blood THC concentrations peaking at 1 h (Schlienz et al. 2020). The delay of drug effect when orally ingesting THC and duration of effect are important considerations for patients being treated with medical cannabis. Oral cannabis products (e.g., edibles) are responsible for the majority of emergency room visits related to cannabis intoxication, and understanding when and how long to expect a drug effect may help prevent accidental intoxication (Hudak et al. 2015; Barrus et al. 2016; Monte et al. 2019).

### Conservative THC dosing and titration

The conservative protocol was developed to be lower and slower than routine with a focus on prevention of side effects and creating comfort with medical cannabis. The initiating and titrating doses of THC are different between the conservative and routine dosing and administration protocols as there may be concern with the psychotropic effects of THC. Our Delphi process led to agreement that 1 mg THC should be considered as the initiating dose, which is consistent with the lowest range set out in the Boehnke and Clauw guidance document (Boehnke and Clauw 2019). The tentative maximum dose of 40 mg THC for conservative regimen is the same as routine. There was discussion on the importance of exercising caution regarding the rate at which THC is titrated, but not the maximum THC dose.

### Medical cannabis safety considerations

The predicted median lethal dose (LD50) for THC is > 1000-fold higher than the effective dose (Thompson et al. 1973; World Health Organization 2012). Unlike opioids, there are limited cannabinoid receptors in the brain stem areas that control vital functions such as respiration (Herkenham et al. 1990). Following oral administration, the LD50 of THC is 800 mg/kg in rats, 3000 mg/kg in dogs, and up to 9000 mg/kg in monkeys. A lethal THC dose for a 70-kg human is therefore estimated at approximately 4000 mg/kg of THC, which is a dose of 280,000 mg THC and likely unachievable with oral consumption, smoking, or vaporization (World Health Organization 2012). Clinicians may feel comfortable with tailoring the medical cannabis treatment regimen knowing that patients are not at a significant overdose death risk. However, cannabis-associated health risks including Cannabis Use Disorder and complications resulting from the psychoactive effects of THC need to be considered, even at low doses (Adam et al. 2020). This concept is important for the operation of motor vehicles, as well as occupational and recreational hazardous activity. When adding THC, the clinician may consider starting the first dose in the evening to limit potential issues with workplace functioning and driving. In addition, THC at night may support sleep quality and many patients with chronic pain suffer from sleep disturbances. Patients often experience an improvement in function as a result of improved sleep quality when treated with medical cannabis (Sanford et al. 2008; Bachhuber et al. 2019). However, the role of medical cannabis and sleep is currently being tested in a placebo-controlled randomized control trial (Suraev et al. 2020).

## Conclusions

In summary, this modified Delphi process, led by global experts in the field of medical cannabis/cannabinoid medicine, resulted in the development of three protocols for the dosing and administration of medical cannabis to treat chronic pain. We hope that these recommendations will support clinicians and patients in achieving safe and effective dosing and administration of medical cannabis. Future randomized control trials examining the safety and efficacy of medical cannabis compared against current standards of care will be required to elucidate whether the developed protocols result in improved patient outcomes. The recommendations provided will be updated as new clinical trial evidence becomes available to inform on the type of dosing and mode of administration of medical cannabis for the treatment of chronic pain.

## Supplementary Information

**Additional file 1.** Practice patterns survey.

**Additional file 2.** First pre vote results.

**Additional file 3.** Second pre vote results.

**Additional file 4.** First virtual meeting voting results.

**Additional file 5.** Second virtual meeting results.

**Additional file 6.** Clinical experience and COI disclosures.

## Abbreviations

CBD: Cannabidiol
THC: Δ-9-Tetrahydrocannabinol

## References

[CR1] Abrams DI, Couey P, Shade SB, Kelly ME, Benowitz NL (2011). Cannabinoid-opioid interaction in chronic pain. Clin Pharmacol Ther.

[CR2] Abuhasira R, Schleider LBL, Mechoulam R, Novack V (2018). Epidemiological characteristics, safety and efficacy of medical cannabis in the elderly. Eur J Intern Med.

[CR3] Adam KCS, Doss MK, Pabon E, Vogel EK, de Wit H (2020). Δ9-Tetrahydrocannabinol (THC) impairs visual working memory performance: a randomized crossover trial. Neuropsychopharmacology..

[CR4] Allan GM, Jamil C, Danielle R (2018). Simplified guideline for prescribing medical cannabinoids in primary care. Can Fam Physician.

[CR5] Andreae MH, Carter GM, Shaparin N, Suslov K, Ellis RJ, Ware MA, Abrams DI, Prasad H, Wilsey B, Indyk D, Johnson M, Sacks HS (2015). Inhaled cannabis for chronic neuropathic pain: a meta-analysis of individual patient data. J Pain.

[CR6] Antoniou T, Bodkin J, Ho JMW (2020). Drug interactions with cannabinoids. CMAJ.

[CR7] Arain M, Haque M, Johal L, Mathur P, Nel W, Rais A, Sandhu R, Sharma S (2013). Maturation of the adolescent brain. Neuropsychiatr Dis Treat.

[CR8] Atalay S, Jarocka-karpowicz I, Skrzydlewskas E (2020). Antioxidative and anti-inflammatory properties of cannabidiol. Antioxidants.

[CR9] Azcarate PM, Zhang AJ, Keyhani S, Steigerwald S, Ishida JH, Cohen BE (2020). Medical reasons for marijuana use, forms of use, and patient perception of physician attitudes among the US population. J Gen Intern Med.

[CR10] Bachhuber M, Arnsten JH, Wurm G (2019). Use of cannabis to relieve pain and promote sleep by customers at an adult use dispensary. J Psychoactive Drugs.

[CR11] Balachandran P, Elsohly M, Hill KP. Cannabidiol interactions with medications, illicit substances, and alcohol: a comprehensive review. J Gen Intern Med. 2021. 10.1007/s11606-020-06504-8. 10.1007/s11606-020-06504-8 PMC8298645 33515191

[CR12] Banerjee S, McCormack S. Medical cannabis for the treatment of chronic pain : a review of clinical effectiveness and guidelines. CADTH Canadian Agency Drugs Technol Health Rapid Response Reports. 2019;1:1–43. 31532599

[CR13] Barrus DG, Capogrossi KL, Cates SC, et al. Tasty THC: promises and challenges of cannabis edibles. Research Triangle Park, NC: Methods Rep RTI Press; 2016. 10.3768/rtipress.2016.op.0035.1611. 10.3768/rtipress.2016.op.0035.1611 PMC5260817 28127591

[CR14] Beal JE, Olson R, Laubenstein L, Morales JO, Bellman P, Yangco B, Lefkowitz L, Plasse TF, Shepard KV (1995). Dronabinol as a treatment for anorexia associated with weight loss in patients with AIDS. J Pain Symptom Manag.

[CR15] Boehnke KF, Clauw DJ (2019). Brief commentary: cannabinoid dosing for chronic pain management. Ann Intern Med.

[CR16] Boehnke KF, Gangopadhyay S, Clauw DJ, Haffajee RL (2019). Qualifying conditions of medical cannabis license holders in the United States. Health Aff.

[CR17] Bonn-Miller MO, Loflin MJE, Thomas BF, Marcu JP, Hyke T, Vandrey R (2017). Labeling accuracy of cannabidiol extracts sold online. JAMA.

[CR18] Boyaji S, Merkow J, Elman RNM, Kaye AD, Yong RJ, Urman RD (2020). The role of cannabidiol (CBD) in chronic pain management: an assessment of current evidence. Curr Pain Headache Rep.

[CR19] Canadian Medical Association (2020). What Canadians think about virtual health care.

[CR20] Capano A, Weaver R, Burkman E (2019). Evaluation of the effects of CBD hemp extract on opioid use and quality of life indicators in chronic pain patients: a prospective cohort study. Postgrad Med.

[CR21] Casey BJ, Jones RM, Hare TA (2013). The adolescent brain. Cogn Behav Neurol.

[CR22] Cooper ZD, Bedi G, Ramesh D, Balter R, Comer SD, Haney M (2018). Impact of co-administration of oxycodone and smoked cannabis on analgesia and abuse liability. Neuropsychopharmacology..

[CR23] Corroon J, MacKay D, Dolphin W (2020). Labeling of cannabidiol products: a public health perspective. cannabis cannabinoid. Res.

[CR24] Cox EJ, Maharao N, Patilea-Vrana G, Unadkat JD, Rettie AE, McCune JS, Paine MF (2019). A marijuana-drug interaction primer: Precipitants, pharmacology, and pharmacokinetics. Pharmacol Ther.

[CR25] Crawley A, Schuster B, Lebras M, et al. Navigating cannabinoid choices for chronic neuropathic pain in older adults. Can Fam Physician. 2019;65:807–11. PMC6853369 31722915

[CR26] Dalkey N (1969). An experimental study of group opinion: the Delphi method. Futures..

[CR27] Dalkey N, Helmer O (1963). An experimental application of the DELPHI Method to the Use of Experts. Manag Sci.

[CR28] Diamond IR, Grant RC, Feldman BM, Pencharz PB, Ling SC, Moore AM, Wales PW (2014). Defining consensus: a systematic review recommends methodologic criteria for reporting of Delphi studies. J Clin Epidemiol.

[CR29] Dueñas M, Ojeda B, Salazar A (2016). A review of chronic pain impact on patients, their social environment and the health care system. J Pain Res.

[CR30] Geffrey AL, Pollack SF, Bruno PL, Thiele EA (2015). Drug-drug interaction between clobazam and cannabidiol in children with refractory epilepsy. Epilepsia.

[CR31] Gillessen S, Attard G, Beer TM (2018). Management of patients with advanced prostate cancer: the report of the advanced prostate cancer consensus conference APCCC 2017 [Figure presented]. European Urology.

[CR32] Goods Administration T (2020). Safety of low dose cannabidiol.

[CR33] Grotenhermen F (2003). Pharmacokinetics and pharmacodynamics of cannabinoids. Clin Pharmacokinet.

[CR34] Gulbransen G, Xu W, Arroll B (2020). Cannabidiol prescription in clinical practice: an audit on the first 400 patients in New Zealand. BJGP Open.

[CR35] Habib G, Artul S (2018). Medical cannabis for the treatment of fibromyalgia. J Clin Rheumatol.

[CR36] Haroutounian S, Ratz Y, Ginosar Y, Furmanov K, Saifi F, Meidan R, Davidson E (2016). The effect of medicinal cannabis on pain and quality-of-life outcomes in chronic pain: a prospective open-label study. Clin J Pain.

[CR37] Hasson F, Keeney S, McKenna H (2000). Research guidelines for the Delphi survey technique. J Adv Nurs.

[CR38] Häuser W, Petzke F, Fitzcharles MA (2018). Efficacy, tolerability and safety of cannabis-based medicines for chronic pain management—an overview of systematic reviews. Eur J Pain (United Kingdom).

[CR39] Herkenham M, Lynn AB, Little MD, Johnson MR, Melvin LS, de Costa BR, Rice KC (1990). Cannabinoid receptor localization in brain. Proc Natl Acad Sci U S A.

[CR40] Hudak M, Severn D, Nordstrom K (2015). Edible cannabis-induced psychosis: Intoxication and beyond. Am J Psychiatry.

[CR41] Huestis MA (2007). Human cannabinoid pharmacokinetics. Chem Biodivers.

[CR42] Hylands-White N, Duarte RV, Raphael JH (2017). An overview of treatment approaches for chronic pain management. Rheumatol Int.

[CR43] Johal H, Devji T, Chang Y, et al. Cannabinoids in chronic non-cancer pain: a systematic review and meta-analysis. Clin Med Insights Arthritis Musculoskelet Disord. 2020;13. 10.1177/1179544120906461 PMC7031792 32127750

[CR44] Kazui M, Nishiya Y, Ishizuka, T, et al. Identification of the Human Cytochrome P450 Enzymes Involved in the Two Oxidative Steps in the Bioactivation of Clopidogrel to Its Pharmacologically Active Metabolite. Drug Metab Dispos. 2010;38(1):92–99. 10.1124/dmd.109.029132. 10.1124/dmd.109.029132 19812348

[CR45] King BA, Jones CM, Baldwin GT, Briss PA (2020). The evali and youth vaping epidemics—implications for public health. N Engl J Med.

[CR46] Kosiba JD, Maisto SA, Ditre JW (2019). Patient-reported use of medical cannabis for pain, anxiety, and depression symptoms: Systematic review and meta-analysis. Soc Sci Med.

[CR47] Lachenmeier DW, Habel S, Fischer B, Herbi F, Zerbe Y, Bock V, Rajcic de Rezende T, Walch SG, Sproll C (2020). Are side effects of cannabidiol (CBD) products caused by tetrahydrocannabinol (THC) contamination?. F1000Research.

[CR48] Larsen C, Shahinas J (2020). Dosage, efficacy and safety of cannabidiol administration in adults: a systematic review of human trials. J Clin Med Res.

[CR49] Lattanzi S, Brigo F, Trinka E, Zaccara G, Cagnetti C, del Giovane C, Silvestrini M (2018). Efficacy and safety of cannabidiol in epilepsy: a systematic review and meta-analysis. Drugs.

[CR50] Layden JE, Ghinai I, Pray I, Kimball A, Layer M, Tenforde MW, Navon L, Hoots B, Salvatore PP, Elderbrook M, Haupt T, Kanne J, Patel MT, Saathoff-Huber L, King BA, Schier JG, Mikosz CA, Meiman J (2019). Pulmonary illness related to e-cigarette use in Illinois and Wisconsin—preliminary report. N Engl J Med.

[CR51] Leino AD, Emoto C, Fukuda T, Privitera M, Vinks AA, Alloway RR (2019). Evidence of a clinically significant drug-drug interaction between cannabidiol and tacrolimus. Am J Transplant.

[CR52] Lintzeris N, Lintzeris N, Mills L (2020). Medical cannabis use in the Australian community following introduction of legal access: The 2018-2019 Online Cross-Sectional Cannabis as Medicine Survey (CAMS-18). Harm Reduct J.

[CR53] Longo R, Oudshoorn A, Befus D (2020). Cannabis for chronic pain: a rapid systematic review of randomized control trials. Pain Manag Nurs.

[CR54] MacCallum CA, Russo EB (2018). Practical considerations in medical cannabis administration and dosing. Eur J Intern Med.

[CR55] Maher DP, Carr DB, Hill K, McGeeney B, Weed V, Jackson WC, DiBenedetto DJ, Moriarty EM, Kulich RJ (2019). Cannabis for the treatment of chronic pain in the era of an opioid epidemic: a symposium-based review of sociomedical science. Pain Med (United States).

[CR56] Mlost J, Bryk M, Starowicz K (2020). Cannabidiol for pain treatment: focus on pharmacology and mechanism of action. Int J Mol Sci.

[CR57] Monte AA, Shelton SK, Mills E, Saben J, Hopkinson A, Sonn B, Devivo M, Chang T, Fox J, Brevik C, Williamson K, Abbott D (2019). Acute illness associated with cannabis use, by route of exposure an observational study. Ann Intern Med.

[CR58] Montero-Oleas N, Arevalo-Rodriguez I, Nuñez-González S, Viteri-García A, Simancas-Racines D (2020). Therapeutic use of cannabis and cannabinoids: an evidence mapping and appraisal of systematic reviews. BMC Complement Med Ther.

[CR59] Mücke M, Phillips T, Radbruch L, et al. Cannabis-based medicines for chronic neuropathic pain in adults. Cochrane Database Syst Rev. 2018;3(3):CD012182. 10.1002/14651858.CD012182.pub2. 10.1002/14651858.CD012182.pub2 PMC6494210 29513392

[CR60] National Academies of Sciences (2017). The health effects of cannabis and cannabinoids.

[CR61] Nguyen JD, Grant Y, Kerr TM, Gutierrez A, Cole M, Taffe MA (2018). Tolerance to hypothermic and antinoceptive effects of ∆9-tetrahydrocannabinol (THC) vapor inhalation in rats. Pharmacol Biochem Behav.

[CR62] Okusanya BO, Asaolu IO, Ehiri JE, Kimaru LJ, Okechukwu A, Rosales C (2020). Medical cannabis for the reduction of opioid dosage in the treatment of non-cancer chronic pain: A systematic review. Syst Rev.

[CR63] Oude Voshaar MAH, Das Gupta Z, Bijlsma JWJ, Boonen A, Chau J, Courvoisier DS, Curtis JR, Ellis B, Ernestam S, Gossec L, Hale C, Hornjeff J, Leung KYY, Lidar M, Mease P, Michaud K, Mody GM, Ndosi M, Opava CH, Pinheiro GRC, Salt M, Soriano ER, Taylor WJ, Voshaar MJH, Weel AEAM, Wit M, Wulffraat N, Laar MAFJ, Vonkeman HE (2019). International consortium for health outcome measurement set of outcomes that matter to people living with inflammatory arthritis: consensus from an international working group. Arthritis Care Res.

[CR64] Owen KP, Sutter ME, Albertson TE (2014). Marijuana: respiratory tract effects. Clin Rev Allergy Immunol.

[CR65] Reiman A, Welty M, Solomon P (2017). Cannabis as a substitute for opioid-based pain medication: patient self-report. Cannabis Cannabinoid Res.

[CR66] Rod K (2019). A pilot study of a medical cannabis—opioid reduction program. Am J Psychiatry Neurosci.

[CR67] Russo EB (2007). History of cannabis and its preparations in saga, science, and sobriquet. Chem Biodivers.

[CR68] Saad F, Canil C, Finelli A, Hotte SJ, Malone S, Shayegan B, et al. A Canadian consensus forum on the management of patients with advanced prostate cancer. Can Urol Assoc J. 2019;14(4). 10.5489/cuaj.6082. 10.5489/cuaj.6082 PMC7124178 31702544

[CR69] Safakish R, Ko G, Salimpour V, Hendin B, Sohanpal I, Loheswaran G, Yoon SYR (2020). Medical cannabis for the management of pain and quality of life in chronic pain patients: a prospective observational study. Pain Med.

[CR70] Sagy I, Bar-Lev Schleider L, Abu-Shakra M, Novack V (2019). Safety and efficacy of medical cannabis in fibromyalgia. J Clin Med.

[CR71] Sanford AE, Castillo E, Gannon RL (2008). Cannabinoids and hamster circadian activity rhythms. Brain Res.

[CR72] Sangmo L, Braune T, Liu B, Wang L, Zhang L, Sosnoff CS, Blount BC, Wilson KM (2020). Secondhand marijuana exposure in a convenience sample of young children in New York City. Pediatr Res.

[CR73] Schlienz NJ, Spindle TR, Cone EJ, Herrmann ES, Bigelow GE, Mitchell JM, Flegel R, LoDico C, Vandrey R (2020). Pharmacodynamic dose effects of oral cannabis ingestion in healthy adults who infrequently use cannabis. Drug Alcohol Depend.

[CR74] Sihota A, Smith BK, Ahmed SA, Bell A, Blain A, Clarke H, et al. Consensus-based recommendations for titrating cannabinoids and tapering opioids for chronic pain control. Int J Clin Pract. 2020. 10.1111/ijcp.13871. 10.1111/ijcp.13871 PMC8365704 33249713

[CR75] Skelley JW, Deas CM, Curren Z, Ennis J (2020). Use of cannabidiol in anxiety and anxiety-related disorders. J Am Pharm Assoc.

[CR76] Stockings E, Campbell G, Hall WD (2018). Cannabis and cannabinoids for the treatment of people with chronic noncancer pain conditions: a systematic review and meta-analysis of controlled and observational studies.

[CR77] Suraev A, Grunstein RR, Marshall NS, D'Rozario AL, Gordon CJ, Bartlett DJ, Wong K, Yee BJ, Vandrey R, Irwin C, Arnold JC, McGregor IS, Hoyos CM (2020). Cannabidiol (CBD) and Δ ^9^ -tetrahydrocannabinol (THC) for chronic insomnia disorder (‘CANSLEEP’ trial): protocol for a randomised, placebo-controlled, double-blinded, proof-of-concept trial. BMJ Open.

[CR78] Taha T, Meiri D, Talhamy S, Wollner M, Peer A, Bar-Sela G (2019). Cannabis impacts tumor response rate to nivolumab in patients with advanced malignancies. Oncologist.

[CR79] Tashkin DP (2013). Effects of marijuana smoking on the lung. Ann Am Thorac Soc.

[CR80] Taylor L, Gidal B, Blakey G, Tayo B, Morrison G (2018). A phase I, randomized, double-blind, placebo-controlled, single ascending dose, multiple dose, and food effect trial of the safety, tolerability and pharmacokinetics of highly purified cannabidiol in healthy subjects. CNS Drugs.

[CR81] Thompson GR, Rosenkrantz H, Schaeppi UH, Braude MC (1973). Comparison of acute oral toxicity of cannabinoids in rats, dogs and monkeys. Toxicol Appl Pharmacol.

[CR82] Volkow ND, Baler RD, Compton WM, Weiss SRB (2014). Adverse health effects of marijuana use. N Engl J Med.

[CR83] Vos T, Abajobir AA, Abbafati C (2017). Global, regional, and national incidence, prevalence, and years lived with disability for 328 diseases and injuries for 195 countries, 1990-2016: A systematic analysis for the Global Burden of Disease Study 2016. Lancet.

[CR84] Wallace MS, Marcotte TD, Atkinson JH, Padovano HT, Bonn-Miller M (2020). A secondary analysis from a randomized trial on the effect of plasma tetrahydrocannabinol levels on pain reduction in painful diabetic peripheral neuropathy. J Pain.

[CR85] Ware MA, Wang T, Shapiro S, Robinson A, Ducruet T, Huynh T, et al. Smoked cannabis for chronic neuropathic pain: A randomized controlled trial. CMAJ. 2010;182. 10.1503/cmaj.091414. 10.1503/cmaj.091414 PMC2950205 20805210

[CR86] Whiting PF, Wolff RF, Deshpande S, di Nisio M, Duffy S, Hernandez AV, Keurentjes JC, Lang S, Misso K, Ryder S, Schmidlkofer S, Westwood M, Kleijnen J (2015). Cannabinoids for medical use: a systematic review and meta-analysis. JAMA.

[CR87] Wilkerson JL, Schulze DR, McMahon LR (2019). Tolerance and dependence to Δ 9 -tetrahydrocannabinol in rhesus monkeys: Activity assessments. PLoS One.

[CR88] Wong SSC, Chan WS, Cheung CW (2020). Analgesic effects of cannabinoids for chronic non-cancer pain: a systematic review and meta-analysis with meta-regression. J NeuroImmune Pharmacol.

[CR89] World Health Organization (2012). WHO expert committee on drug dependence.

[CR90] Yamaori S, Koeda K, Kushihara M, Hada Y, Yamamoto I, Watanabe K (2012). Comparison in the in vitro inhibitory effects of major phytocannabinoids and polycyclic aromatic hydrocarbons contained in marijuana smoke on cytochrome P450 2C9 activity. Drug Metab Pharmacokinet.

[CR91] Yamreudeewong W, Wong HK, Brausch LM, Pulley KR (2009). Probable interaction between warfarin and marijuana smoking. Ann Pharmacother.

[CR92] Zhu HJ, Wang JS, Markowitz JS, Donovan JL, Gibson BB, Gefroh HA, DeVane CL (2006). Characterization of P-glycoprotein inhibition by major cannabinoids from marijuana. J Pharmacol Exp Ther.

