# Supplementary material for: Consensus recommendations on dosing and administration of medical cannabis to treat chronic pain: results of a modified Delphi process
Source: J Cannabis Res. 2021 Jul 2;3:22. doi: 10.1186/s42238-021-00073-1 (PMC8252988; doi:10.1186/s42238-021-00073-1)
Supplement: Supplementary file 1 — Additional file 1. Practice patterns survey. [file 42238_2021_73_MOESM1_ESM.docx]

**Dosing and Administration of Medical Cannabis: Physician Survey**

1. What is your speciality?
2. Do you have access to prescribing medical cannabis?
3. About how many patients have you prescribed or managed on medical cannabis?
4. How many years of experience do you have prescribing medical cannabis?
5. If applicable, how many years have you been educating patients and physicians about medical cannabis?
6. Which types of chronic pain do you typically treat with medical cannabis?
7. What patient conditions or medications might cause you to avoid prescribing/authorizing medical cannabis for chronic pain?
8. Based on your clinical practice, what type of medical cannabis product is most effective for treating chronic non-cancer pain; THC-dominant, balanced, or CBD-dominant?
9. What dose of THC and/or CBD do you typically initiate with?
10. How do you titrate THC and CBD?
11. Based on your clinical practice, what is the therapeutic dose of THC and CBD in patients with chronic non-cancer pain?
12. Is your dosing and titration procedure altered across the different types of chronic non-cancer pain? If so, how?
13. What are the safety considerations when treating with medical cannabis?
14. How should patients taking medical cannabis be monitored?
15. What does clinical treatment success look like?
