# Supplementary material for: Consensus recommendations on dosing and administration of medical cannabis to treat chronic pain: results of a modified Delphi process
Source: J Cannabis Res. 2021 Jul 2;3:22. doi: 10.1186/s42238-021-00073-1 (PMC8252988; doi:10.1186/s42238-021-00073-1)
Supplement: Supplementary file 2 — Additional file 2. First pre vote results. [file 42238_2021_73_MOESM2_ESM.pdf]

Consensus Voting Round 1 Results

Consensus Reached with ≥75%

n=16

Rationale for using medical cannabis in patients with chronic pain, include:

1	#	Question	Strongly Disagree	Disagree	Neutral	Agree	Strongly agree	Abstain	Total	Strongly Disagree/ Disagree	Strongly Agree/ Agree	1
	1	Reduction in pain intensity	0.0%	0.0%	5.6%	16.7%	77.8%	0.0%	18	0.0%	94.5%	
	2	Reduction in frequency of pain	0.0%	5.6%	5.6%	44.4%	44.4%	0.0%	18	5.6%	88.9%	
	3	Reduction in breakthrough pain frequency	0.0%	11.1%	5.6%	50.0%	33.3%	0.0%	18	11.1%	83.3%	
	4	Improvement in patient physical function	0.0%	0.0%	5.6%	16.7%	72.2%	5.6%	18	0.0%	88.9%	
	5	Improvement in patient activities of daily living	0.0%	0.0%	5.6%	11.1%	77.8%	5.6%	18	0.0%	88.9%	
	6	Improvement in mental health symptoms (e.g. distress, anxiety, depressed mood)	0.0%	0.0%	11.1%	44.4%	38.9%	5.6%	18	0.0%	83.3%	
	7	Poor response to current treatment	0.0%	0.0%	0.0%	27.8%	72.2%	0.0%	18	0.0%	100.0%	
	8	Management of patient's pain and comorbidities (e.g. insomnia, depression)	0.0%	0.0%	0.0%	44.4%	50.0%	5.6%	18	0.0%	94.4%	
	9	Reduction in opioid use	0.0%	0.0%	0.0%	38.9%	61.1%	0.0%	18	0.0%	100.0%	
	10	Reduction in other analgesic use (e.g. acetaminophen (paracetamol), NSAIDs)	0.0%	11.1%	5.6%	50.0%	33.3%	0.0%	18	11.1%	83.3%	
	11	Reduction in anti-depressant use	0.0%	22.2%	33.3%	27.8%	16.7%	0.0%	18	22.2%	44.5%	
	12	Reduction in anti-epileptic use	0.0%	22.2%	22.2%	38.9%	16.7%	0.0%	18	22.2%	55.6%	
	13	Reduction in benzodiazepine use	0.0%	0.0%	0.0%	50.0%	50.0%	0.0%	18	0.0%	100.0%	
	14	Reduction in substance use (e.g. alcohol, illicit drug)	0.0%	0.0%	5.6%	33.3%	61.1%	0.0%	18	0.0%	94.4%	
	15	Reduction of adverse effects and risks from the patientâ€™s other pharmacotherapy	0.0%	0.0%	5.6%	55.6%	38.9%	0.0%	18	0.0%	94.5%	
	16	Patient preference for cannabis use	5.6%	5.6%	22.2%	50.0%	16.7%	0.0%	18	11.1%	66.7%	

Medical cannabis is likely effective for

1	#	Question	Strongly Disagree	Disagree	Neutral	Agree	Strongly agree	Abstain	Total	Strongly Disagree/	Strongly Agree/	1
	1	Chronic inflammatory pain (e.g. osteoarthritis, rheumatoid arthritis)	0.0%	0.0%	5.6%	66.7%	27.8%	0.0%	18	0.0%	94.5%	
	2	Chronic nociceptive pain with supraspinal sensitisation	0.0%	0.0%	0.0%	66.7%	27.8%	5.6%	18	0.0%	94.5%	
	3	Chronic nociceptive pain without supraspinal sensitisation	0.0%	5.6%	22.2%	50.0%	16.7%	5.6%	18	5.6%	66.7%	
	4	Chronic neuropathic pain (e.g. diabetic neuropathy)	0.0%	0.0%	0.0%	22.2%	72.2%	5.6%	18	0.0%	94.4%	
	5	Chronic visceral pain (e.g. pelvic pain)	0.0%	0.0%	5.6%	72.2%	22.2%	0.0%	18	0.0%	94.4%	
	6	Chronic traumatic pain	0.0%	0.0%	11.1%	55.6%	27.8%	5.6%	18	0.0%	83.3%	
	7	Chronic cancer pain	0.0%	0.0%	5.6%	38.9%	55.6%	0.0%	18	0.0%	94.5%	
	8	Palliative Pain	0.0%	0.0%	0.0%	44.4%	50.0%	5.6%	18	0.0%	94.4%	
	9	Breakthrough pain	0.0%	11.1%	11.1%	55.6%	22.2%	0.0%	18	11.1%	77.8%	

The minimum age for use of CBD in patients with chronic pain is:

1	#	Answer	%	1
	1	30 years of age	0.0%	
	2	25 years of age	16.7%	
	3	21 years of age	0.0%	
	4	18 years of age	16.7%	
	5	No minimum age	66.7%	
	6	Abstain	0.0%	
		Total	100.0%	

The minimum age for use of THC in patients with chronic pain is:

1	#	Answer	%
	1	30 years of age	0.0%
	2	25 years of age	27.8%
	3	21 years of age	27.8%
	4	18 years of age	16.7%
	5	No minimum age	27.8%
	6	Abstain	0.0%

	Total	100.0%

The maximum age for use of CBD in patients with chronic pain is:

1 #	Answer	%
1	60 years of age	0.0%
2	70 years of age	0.0%
3	80 years of age	0.0%
4	90 years of age	0.0%
5	No maximum age	100.0%
6	Abstain	0.0%
	Total	100.0%

The maximum age for use of THC in patients with chronic pain is:

1 #	Answer	%
1	60 years of age	0.0%
2	70 years of age	0.0%
3	80 years of age	5.6%
4	90 years of age	0.0%
5	No maximum age	94.4%
6	Abstain	0.0%
	Total	100.0%

Medical cannabis should be avoided in patients with chronic pain with the following conditions:

1 #	Question	Strongly Disagree	Disagree	Neutral	Agree	Strongly agree	Abstain	Total	Strongly Disagree/ Disagree	Strongly Agree/ Agree
1	A patient with a psychotic disorder	0.0%	11.1%	0.0%	33.3%	55.6%	0.0%	18	11.1%	88.9%
2	A patient with a family history of psychotic disorders	11.1%	16.7%	33.3%	33.3%	5.6%	0.0%	18	27.8%	38.9%
3	A patient with mood disorder	16.7%	44.4%	22.2%	16.7%	0.0%	0.0%	18	61.1%	16.7%
4	A patient with personality disorder	5.6%	38.9%	22.2%	16.7%	16.7%	0.0%	18	44.5%	33.3%
5	A patient with substance abuse disorder	16.7%	22.2%	22.2%	16.7%	22.2%	0.0%	18	38.9%	38.9%
6	A patient who is a heavy user of alcohol	16.7%	22.2%	22.2%	27.8%	11.1%	0.0%	18	38.9%	38.9%
7	A patient who is taking high doses of prescribed opioids	50.0%	33.3%	11.1%	5.6%	0.0%	0.0%	18	83.3%	5.6%
8	A patient who is taking high doses benzodiazepines or other sedating medications prescribed or over the counter	27.8%	38.9%	22.2%	5.6%	5.6%	0.0%	18	66.7%	11.1%
9	A patient with severe cardiovascular disease	5.6%	22.2%	22.2%	27.8%	22.2%	0.0%	18	27.8%	50.0%
10	A patient who smokes tobacco	44.4%	44.4%	11.1%	0.0%	0.0%	0.0%	18	88.9%	0.0%
11	A patient with severe pulmonary disease for inhaled cannabis	0.0%	27.8%	11.1%	33.3%	27.8%	0.0%	18	27.8%	61.1%
12	A patient with severe renal disease	27.8%	38.9%	16.7%	16.7%	0.0%	0.0%	18	66.7%	16.7%
13	A patient with severe hepatic disease	16.7%	27.8%	16.7%	38.9%	0.0%	0.0%	18	44.5%	38.9%
14	Pediatric patients	22.2%	27.8%	11.1%	27.8%	11.1%	0.0%	18	50.0%	38.9%
15	Geriatric patients (>65 years)	61.1%	33.3%	0.0%	5.6%	0.0%	0.0%	18	94.4%	5.6%
16	A patient with social instability	11.1%	38.9%	27.8%	22.2%	0.0%	0.0%	18	50.0%	22.2%
17	A patient with immunosuppression	22.2%	38.9%	33.3%	0.0%	5.6%	0.0%	18	61.1%	5.6%
18	A patient who is pregnant or planning to become pregnant	0.0%	5.6%	5.6%	22.2%	61.1%	5.6%	18	5.6%	83.3%
19	A patient who is breastfeeding	0.0%	5.6%	0.0%	27.8%	61.1%	5.6%	18	5.6%	88.9%

Medical cannabis should be avoided in patients with chronic pain being administered the following medications:

1 #	Question	Strongly Disagree	Disagree	Neutral	Agree	Strongly agree	Abstain	Total	Strongly Disagree/ Disagree	Strongly Agree/ Agree
1	Warfarin	5.9%	52.9%	17.7%	11.8%	11.8%	0.0%	17	58.8%	23.5%
2	Direct oral anticoagulants (DOACs)	5.9%	52.9%	11.8%	17.7%	11.8%	0.0%	17	58.8%	29.4%
3	Opioids	50.0%	38.9%	5.6%	0.0%	5.6%	0.0%	18	88.9%	5.6%
4	Opioid agonist treatment (e.g. methadone or buprenorphine)	47.1%	47.1%	5.9%	0.0%	0.0%	0.0%	17	94.1%	0.0%
5	Benzodiazepines	33.3%	55.6%	5.6%	5.6%	0.0%	0.0%	18	88.9%	5.6%
6	Skeletal muscle relaxants	41.2%	47.1%	11.8%	0.0%	0.0%	0.0%	17	88.2%	0.0%
7	Hypnotics	22.2%	61.1%	11.1%	5.6%	0.0%	0.0%	18	83.3%	5.6%
8	Non-steroidal anti-inflammatory drugs (NSAIDs)	44.4%	55.6%	0.0%	0.0%	0.0%	0.0%	18	100.0%	0.0%
9	Antidepressants	38.9%	55.6%	5.6%	0.0%	0.0%	0.0%	18	94.5%	0.0%
10	Anti-psychotics	22.2%	44.4%	22.2%	5.6%	5.6%	0.0%	18	66.7%	11.1%
11	Corticosteroids	38.9%	50.0%	11.1%	0.0%	0.0%	0.0%	18	88.9%	0.0%
12	Anti-hypercholesterolemic agents	44.4%	50.0%	5.6%	0.0%	0.0%	0.0%	18	94.4%	0.0%
13	Antihypertensive agents	38.9%	55.6%	5.6%	0.0%	0.0%	0.0%	18	94.5%	0.0%

14	Antimicrobial agents	38.9%	50.0%	5.6%	0.0%	0.0%	5.6%	18	88.9%	0.0%
15	Anti-epileptic agents (including clobazam)	16.7%	61.1%	16.7%	5.6%	0.0%	0.0%	18	77.8%	5.6%
16	Antihyperglycemic agents	38.9%	55.6%	0.0%	0.0%	0.0%	5.6%	18	94.5%	0.0%
17	Cyclosporine or tacrolimus	22.2%	55.6%	11.1%	0.0%	5.6%	5.6%	18	77.8%	5.6%
18	Cancer chemotherapy	44.4%	38.9%	11.1%	0.0%	0.0%	5.6%	18	83.3%	0.0%
19	Disease modifying synthetic anti-rheumatic drugs (DMARDs)	38.9%	44.4%	11.1%	0.0%	0.0%	5.6%	18	83.3%	0.0%
20	Biologic therapies for rheumatoid arthritis, inflammatory bowel disease, etc.	38.9%	38.9%	16.7%	0.0%	0.0%	5.6%	18	77.8%	0.0%
21	Immunotherapy agents for oncology (e.g. checkpoint inhibitors)	27.8%	22.2%	27.8%	11.1%	0.0%	11.1%	18	50.0%	11.1%

The starting THC:CBD ratio for most patients with chronic pain should be:

	#	Question	Strongly Disagree	Disagree	Neutral	Agree	Strongly agree	Abstain	Total	Strongly Disagree/ Disagree	Strongly Agree/ Agree
1											
	1	CBD predominant	5.9%	5.9%	5.9%	58.8%	23.5%	0.0%	17	11.8%	82.4%
	2	Balanced THC:CBD	5.6%	0.0%	16.7%	44.4%	33.3%	0.0%	18	5.6%	77.8%
	3	THC predominant	12.5%	50.0%	6.3%	18.8%	12.5%	0.0%	16	62.5%	31.3%

The starting CBD daily dose for most patients is: (total amount in 24 hours)

	#	Answer	%
1			
	1	5 mg	22.2%
	2	10 mg	50.0%
	3	20 mg	5.6%
	4	40 mg	0.0%
	5	50 mg	5.6%
	6	> 50 mg	16.7%
	7	Abstain	0.0%
		Total	100.0%

The recommended starting dosing frequency for CBD is: (Daily dose divided into this frequency)

	#	Answer	%
1			
	1	Once daily	33.3%
	2	Twice daily	66.7%
	3	Three times daily	0.0%
	4	Four times daily	0.0%
	5	> 4 times daily	0.0%
	6	Abstain	0.0%
		Total	100.0%

The recommended CBD titration amount for most patients is:

	#	Answer	%
1			
	1	1 mg	5.6%
	2	5 mg	33.3%
	3	10 mg	38.9%
	4	20 mg	11.1%
	5	> 20 mg	11.1%
	6	Abstain	0.0%
		Total	100.0%

The recommended CBD up-titration frequency for most patients is:

	#	Answer	%
1			
	1	Daily	11.1%
	2	Every other day	5.6%
	3	2-3 times a week	38.9%
	4	Once a week	38.9%
	5	every 2 weeks	5.6%
	6	Abstain	0.0%
		Total	100.0%

If starting with a CBD predominant variety, THC for most patients should be introduced:

#	Question	Strongly Disagree	Disagree	Neutral	Agree	Strongly agree	Abstain	Total	Strongly Disagree/ Disagree	Strongly Agree/ Agree
1	If needed, add THC at bedtime after one week regardless of CBD dose	5.9%	47.1%	29.4%	17.7%	0.0%	0.0%	17	52.9%	17.7%
2	If needed, add THC at bedtime after two weeks regardless of CBD dose	5.9%	47.1%	29.4%	17.7%	0.0%	0.0%	17	52.9%	17.7%
3	If needed, add THC at bedtime once patient is taking > 20mg of CBD	5.6%	11.1%	22.2%	38.9%	16.7%	5.6%	18	16.7%	55.6%
4	If needed, add THC at bedtime once patient is taking > 40mg of CBD	5.9%	23.5%	23.5%	23.5%	23.5%	0.0%	17	29.4%	47.1%
5	If needed add THC at bedtime when the patient is not the maximally tolerated or afforded CBD dose	0.0%	25.0%	18.8%	25.0%	25.0%	6.3%	16	25.0%	50.0%
6	I do not recommend starting with CBD predominant before initiating THC for most patients with chronic pain	27.8%	22.2%	16.7%	5.6%	22.2%	5.6%	18	50.0%	27.8%

The recommended starting THC daily dose for most patients: (amount for 24 hours)

#	Answer	%
1	1 mg	27.8%
2	2.5 mg	66.7%
3	5 mg	5.6%
4	10 mg	0.0%
5	Abstain	0.0%
	Total	100.0%

The recommended THC titration amountfor most patients is:

#	Answer	%
1	1 mg	27.8%
2	2.5 mg	72.2%
3	5 mg	0.0%
4	10 mg	0.0%
5	Abstain	0.0%
	Total	100.0%

The recommended THC up-titration frequency for most patients is:

#	Answer	%
1	Daily	11.1%
2	Every other day	5.6%
3	2-3 times a week	44.4%
4	Once a week	27.8%
5	Every 2 weeks	11.1%
6	Abstain	0.0%
	Total	100.0%

The recommended maximum daily dose of CBD is:

#	Answer	%
1	50 mg	5.9%
2	100 mg	5.9%
3	300 mg	29.4%
4	600 mg	11.8%
5	No maximum daily dose	41.2%
6	Abstain	5.9%
	Total	100.0%

The recommended maximum daily dose of THC is:

#	Answer	%
1	10 mg	0.0%
2	20 mg	23.5%
3	40 mg	41.2%
4	80 mg	17.7%
5	No maximum daily dose	17.7%
6	Abstain	0.0%
	Total	100.0%

The patients and conditions that should be considered for rapid/aggressive medical cannabis dosing and titration are:

1 #	Question	Strongly Disagree	Disagree	Neutral	Agree	Strongly agree	Abstain	Total	Strongly Disagree/ Disagree	Strongly Agree/ Agree
1	The patient is cannabis experienced (medical or recreational)	0.0%	0.0%	11.1%	55.6%	33.3%	0.0%	18	0.0%	88.9%
2	The patient is taking > 90 mg morphine equivalent dose (MED)	5.6%	33.3%	16.7%	38.9%	5.6%	0.0%	18	38.9%	44.5%
3	The patient is suffering for severe pain or with severe functional impairment	0.0%	16.7%	0.0%	72.2%	11.1%	0.0%	18	16.7%	83.3%
4	The patient who is currently using opioids, gabapentinoids or SNRIs	0.0%	55.6%	27.8%	11.1%	5.6%	0.0%	18	55.6%	16.7%
5	The patient who is has been unsuccessfully treated with opioids, gabapentinoids or SNRIs	0.0%	38.9%	27.8%	22.2%	11.1%	0.0%	18	38.9%	33.3%
6	The patient is experiencing primarily neuropathic pain	0.0%	50.0%	16.7%	16.7%	16.7%	0.0%	18	50.0%	33.3%
7	Palliative care or end of life	0.0%	5.6%	0.0%	55.6%	38.9%	0.0%	18	5.6%	94.5%
8	Lack of efficacy at low/moderate cannabis doses	0.0%	11.1%	16.7%	38.9%	33.3%	0.0%	18	11.1%	72.2%

The recommended starting THC:CBD ratio for patients with chronic pain requiring rapid/aggressive dosing and titration is:

1 #	Question	Strongly Disagree	Disagree	Neutral	Agree	Strongly agree	Abstain	Total	Strongly Disagree/ Disagree	Strongly Agree/ Agree
1	CBD predominant	12.5%	25.0%	12.5%	25.0%	25.0%	0.0%	16	37.5%	50.0%
2	Balanced THC:CBD	0.0%	0.0%	16.7%	55.6%	27.8%	0.0%	18	0.0%	83.3%
3	THC predominant	0.0%	41.2%	11.8%	29.4%	17.7%	0.0%	17	41.2%	47.1%

The recommended starting THC daily dose for patients with chronic pain requiring rapid/aggressive dosing and titration: (amount for 24 hours)

1 #	Answer	%
1	1 mg	11.1%
2	2.5 mg	38.9%
3	5 mg	38.9%
4	10 mg	5.6%
5	Abstain	5.6%
	Total	100.0%

The recommended starting dosing frequency of THC for patients with chronic pain requiring rapid/aggressive dosing and titration: (Daily dose divided into this frequency)

1 #	Answer	%
1	Once daily	22.2%
2	Twice daily	55.6%
3	Three times daily	16.7%
4	Four times daily	0.0%
5	> 4 times daily	0.0%
6	Abstain	5.6%
	Total	100.0%

The recommended THC up-titration frequency for patients with chronic pain requiring rapid/aggressive dosing and titration:

1 #	Answer	%
1	Daily	27.8%
2	Every other day	22.2%
3	2-3 times a week	38.9%
4	Once a week	5.6%
5	Every 2 weeks	0.0%
6	Abstain	5.6%
	Total	100.0%

The recommended maximum daily dose of THC for patients with chronic pain requiring rapid/aggressive dosing and titration:

1 #	Answer	%
1	10 mg	0.0%
2	20 mg	5.6%
3	40 mg	38.9%
4	80 mg	16.7%
5	No maximum daily dose	33.3%
6	Abstain	5.6%
	Total	100.0%

The starting CBD daily dose for patients with chronic pain requiring rapid/aggressive dosing and titration: (total amount in 24 hours)

1	#	Answer	%
	1	5 mg	11.1%
	2	10 mg	27.8%
	3	20 mg	33.3%
	4	40 mg	0.0%
	5	50 mg	0.0%
	6	> 50 mg	16.7%
	7	I would start with THC only	11.1%
	8	Abstain	0.0%
		Total	100.0%

The recommended starting dosing frequency of CBD for patients with chronic pain requiring rapid/aggressive dosing and titration: (Daily dose divided into this frequency)

1	#	Answer	%
	1	Once daily	16.7%
	2	Twice daily	55.6%
	3	Three times daily	16.7%
	4	Four times daily	0.0%
	5	> 4 times daily	0.0%
	6	I would start with THC only	5.6%
	7	Abstain	5.6%
		Total	100.0%

The recommended CBD titration amount for patients with chronic pain requiring rapid/aggressive dosing and titration:

1	#	Answer	%
	1	1 mg	0.0%
	2	5 mg	22.2%
	3	10 mg	22.2%
	4	20 mg	33.3%
	5	> 20 mg	5.6%
	6	I would start with THC only	11.1%
	7	Abstain	5.6%
		Total	100.0%

The recommended CBD up-titration frequency

1	#	Answer	%
	1	Daily	33.3%
	2	Every other day	16.7%
	3	2-3 times a week	38.9%
	4	Once a week	5.6%
	5	Every 2 weeks	0.0%
	6	Abstain	5.6%
		Total	100.0%

The recommended maximum daily dose of CBD

1	#	Answer	%
	1	50 mg	0.0%
	2	100 mg	11.1%
	3	300 mg	16.7%
	4	600 mg	11.1%
	5	No maximum daily dose	44.4%
	6	I would start with THC only	11.1%
	7	Abstain	5.6%
		Total	100.0%

The patients and conditions should be considered for conservative medical cannabis dosing and titration:

1	#	Question	Strongly Disagree	Disagree	Neutral	Agree	Strongly agree	Abstain	Total	Strongly Disagree/ Disagree	Strongly Agree/ Agree
	1	Patient > 65 years	0.0%	5.6%	16.7%	66.7%	11.1%	0.0%	18	5.6%	77.8%
	2	Patient > 70 years	0.0%	0.0%	5.9%	64.7%	29.4%	0.0%	17	0.0%	94.1%
	3	Patient > 80 years	0.0%	0.0%	0.0%	11.8%	88.2%	0.0%	17	0.0%	100.0%

4	Patient with clinical frailty	0.0%	0.0%	0.0%	38.9%	61.1%	0.0%	18	0.0%	100.0%
5	Patient with mental health disorders	0.0%	11.1%	5.6%	16.7%	66.7%	0.0%	18	11.1%	83.3%
6	Patient with cardiovascular disorders	0.0%	5.6%	11.1%	38.9%	44.4%	0.0%	18	5.6%	83.3%
7	Patient with complex comorbidities	0.0%	0.0%	5.6%	50.0%	44.4%	0.0%	18	0.0%	94.4%
8	Patient with polypharmacy	0.0%	0.0%	0.0%	72.2%	27.8%	0.0%	18	0.0%	100.0%

The recommended starting THC:CBD ratio for patients with chronic pain requiring conservative dosing and titration is:

	#	Question	Strongly Disagree	Disagree	Neutral	Agree	Strongly agree	Abstain	Total	Strongly Disagree/ Disagree	Strongly Agree/ Agree
1											
	1	CBD predominant	5.6%	11.1%	5.6%	33.3%	44.4%	0.0%	18	16.7%	77.8%
	2	Balanced THC:CBD	11.8%	11.8%	11.8%	58.8%	5.9%	0.0%	17	23.5%	64.7%
	3	THC predominant	17.7%	52.9%	17.7%	0.0%	11.8%	0.0%	17	70.6%	11.8%

The starting CBD daily dose for patients with chronic pain requiring conservative dosing and titration: (total amount in 24 hours)

	#	Answer	%
1			
	1	5 mg	55.6%
	2	10 mg	22.2%
	3	20 mg	5.6%
	4	40 mg	0.0%
	5	50 mg	5.6%
	6	> 50 mg	11.1%
	7	Abstain	0.0%
		Total	100.0%

The recommended the CBD titration amount for patients with chronic pain requiring conservative dosing and titration:

	#	Answer	%
1			
	1	1 mg	22.2%
	2	5 mg	33.3%
	3	10 mg	16.7%
	4	20 mg	5.6%
	5	> 20 mg	22.2%
	6	Abstain	0.0%
		Total	100.0%

The recommended the CBD up-titration frequency

	#	Answer	%
1			
	1	Daily	11.1%
	2	Every other day	5.6%
	3	2-3 times a week	22.2%
	4	Once a week	38.9%
	5	Every 2 weeks	22.2%
	6	Abstain	0.0%
		Total	100.0%

The recommended maximum daily dose of CBD for patients with chronic pain requiring conservative dosing and titration:

	#	Answer	%
1			
	1	50 mg	5.6%
	2	100 mg	22.2%
	3	300 mg	27.8%
	4	600 mg	0.0%
	5	No maximum daily dose	33.3%
	6	Abstain	11.1%
		Total	100.0%

If starting with an CBD predominant variety, THC for patients with chronic pain requiring conservative dosing and titration should be introduced:

	#	Question	Strongly Disagree	Disagree	Neutral	Agree	Strongly agree	Abstain	Total	Strongly Disagree/ Disagree	Strongly Agree/ Agree
1											
	1	If needed, add THC at bedtime after one week regardless of CBD dose	16.7%	44.4%	11.1%	16.7%	11.1%	0.0%	18	61.1%	27.8%

2	If needed, add THC at bedtime after two weeks regardless of CBD dose	11.8%	52.9%	5.9%	17.7%	11.8%	0.0%	17	64.7%	29.4%
3	If needed, add THC at bedtime once patient is taking > 20mg of CBD	11.8%	35.3%	23.5%	23.5%	5.9%	0.0%	17	47.1%	29.4%
4	If needed, add THC at bedtime once patient is taking > 40mg of CBD	5.9%	23.5%	23.5%	47.1%	0.0%	0.0%	17	29.4%	47.1%
5	If needed add THC at bedtime when the patient is not the maximally tolerated or afforded CBD dose	5.9%	11.8%	17.7%	47.1%	11.8%	5.9%	17	17.6%	58.8%
6	I do not recommend starting with CBD predominant before initiating THC for most patients with chronic pain	31.3%	31.3%	6.3%	6.3%	12.5%	12.5%	16	62.5%	18.8%

The recommended starting THC daily dose for patients with chronic pain requiring conservative dosing and titration: (amount for 24 hours)

1 #	Answer	%
1	1 mg	72.2%
2	2.5 mg	27.8%
3	5 mg	0.0%
4	10 mg	0.0%
5	I don't recommend THC in these patients	0.0%
6	Abstain	0.0%
	Total	100.0%

The recommended starting dosing frequency for THC for patients with chronic pain requiring conservative dosing and titration: (Daily dose divided into this frequency)

1 #	Answer	%
1	Once daily	83.3%
2	Twice daily	11.1%
3	Three times daily	5.6%
4	Four times daily	0.0%
5	> 4 times daily	0.0%
6	I don't recommend THC in these patients	0.0%
7	Abstain	0.0%
	Total	100.0%

The recommended THC up-titration frequency for patients with chronic pain requiring conservative dosing and titration:

1 #	Answer	%
1	Daily	5.6%
2	Every other day	5.6%
3	2-3 times a week	27.8%
4	Once a week	38.9%
5	Every 2 weeks	22.2%
6	I don't recommend THC in these patients	0.0%
7	Abstain	0.0%
	Total	100.0%

The recommended maximum daily dose of THC for patients with chronic pain requiring conservative dosing and titration:

1 #	Answer	%
1	10 mg	11.1%
2	20 mg	50.0%
3	40 mg	11.1%
4	80 mg	5.6%
5	No maximum daily dose	22.2%
6	I don't recommend THC in these patients	0.0%
7	Abstain	0.0%
	Total	100.0%

The patients where inhaled cannabis should be considered for chronic pain management.

1 #	Question	Strongly Disagree	Disagree	Neutral	Agree	Strongly agree	Abstain	Total	Strongly Disagree/ Disagree	Strongly Agree/ Agree
1	Patients currently using inhaled cannabis	0.0%	23.5%	11.8%	47.1%	17.7%	0.0%	17	23.5%	64.7%
2	Patients refusing to use oral cannabis extract	11.8%	23.5%	11.8%	47.1%	5.9%	0.0%	17	35.3%	52.9%
3	In countries where cannabis extracts are not available	0.0%	0.0%	11.8%	52.9%	35.3%	0.0%	17	0.0%	88.2%
4	I prefer inhaled cannabis for chronic pain patients	41.2%	29.4%	17.7%	11.8%	0.0%	0.0%	17	70.6%	11.8%
5	I do not recommend inhaled cannabis for chronic pain patients	17.7%	41.2%	0.0%	17.7%	17.7%	5.9%	17	58.8%	35.3%

The recommended starting THC:CBD ratio for patients with chronic pain for inhaled use:

1	#	Question	Strongly Disagree	Disagree	Neutral	Agree	Strongly agree	Abstain	Total	Strongly Disagree/ Disagree	Strongly Agree/ Agree
	1	CBD predominant	18.8%	12.5%	18.8%	31.3%	12.5%	6.3%	16	31.3%	43.8%
	2	Balanced THC:CBD	0.0%	11.8%	23.5%	35.3%	23.5%	5.9%	17	11.8%	58.8%
	3	THC predominant	18.8%	25.0%	18.8%	18.8%	12.5%	6.3%	16	43.8%	31.3%

The starting dose of medical cannabis for inhaled use:

1	#	Answer	%
	1	1 inhalation	47.1%
	2	2 inhalations	29.4%
	3	3 inhalations	0.0%
	4	> 3 inhalations	0.0%
	5	I don't recommend inhaled cannabis	11.8%
	6	Abstain	11.8%
		Total	100.0%

The recommended daily dosing for inhaled cannabis is:

1	#	Answer	%
	1	Once daily when required	5.9%
	2	Twice daily when required	11.8%
	3	Three times daily when required	5.9%
	4	Four times daily when required	17.7%
	5	Every 4 hours when required	29.4%
	6	Every 2 hours when required	5.9%
	7	I don't recommend inhaled cannabis	11.8%
	8	Abstain	11.8%
		Total	100.0%

The recommended up-titration amount for medical cannabis for inhaled use is:

1	#	Answer	%
	1	1 inhalation	58.8%
	2	2 inhalations	11.8%
	3	3 inhalations	0.0%
	4	> 3 inhalations	0.0%
	5	I don't recommend inhaled cannabis	17.7%
	6	Abstain	11.8%
		Total	100.0%

The recommended dosing and administration considerations for treating breakthrough pain with medical cannabis are:

1	#	Question	Strongly Disagree	Disagree	Neutral	Agree	Strongly agree	Abstain	Total	Strongly Disagree/ Disagree	Strongly Agree/ Agree
	1	Clinicians should increase the dose of the same cannabis product to manage breakthrough pain	0.0%	17.7%	29.4%	41.2%	5.9%	5.9%	17	17.7%	47.1%
	2	Clinicians should increase the frequency of use of the same cannabis product to manage breakthrough pain	0.0%	0.0%	11.8%	76.5%	5.9%	5.9%	17	0.0%	82.4%
	3	Clinicians should increase the THC dose to manage the patientâ€™s breakthrough pain	0.0%	11.8%	35.3%	41.2%	5.9%	5.9%	17	11.8%	47.1%
	4	Clinicians should add as needed oral cannabis to manage breakthrough pain	0.0%	35.3%	17.7%	35.3%	5.9%	5.9%	17	35.3%	41.2%
	5	Clinicians should increase the CBD dose to manage the patientâ€™s breakthrough pain	5.9%	35.3%	29.4%	23.5%	0.0%	5.9%	17	41.2%	23.5%
	6	Clinicians should add as needed inhaled cannabis to manage breakthrough pain	0.0%	5.9%	0.0%	58.8%	23.5%	11.8%	17	5.9%	82.4%
	7	Cannabis should not be used for breakthrough pain or distress	52.9%	29.4%	0.0%	5.9%	5.9%	5.9%	17	82.4%	11.8%

The preferred administration format(s) when using medical cannabis for breakthrough pain treatment is:

1	#	Question	Strongly Disagree	Disagree	Neutral	Agree	Strongly agree	Abstain	Total	Strongly Disagree/ Disagree	Strongly Agree/ Agree
	1	Inhaled smoked	50.0%	18.8%	6.3%	12.5%	6.3%	6.3%	16	68.8%	18.8%
	2	Inhaled vaporized (dried flower)	5.9%	5.9%	0.0%	35.3%	41.2%	11.8%	17	11.8%	76.5%
	3	Inhaled vaporized (prefilled vaporizer pen)	5.9%	23.5%	0.0%	29.4%	35.3%	5.9%	17	29.4%	64.7%
	4	Mucosal spray	0.0%	11.8%	23.5%	47.1%	11.8%	5.9%	17	11.8%	58.8%
	5	Oral oil	0.0%	41.2%	23.5%	23.5%	5.9%	5.9%	17	41.2%	29.4%
	6	Oral soft gels/capsule	5.9%	52.9%	17.7%	11.8%	5.9%	5.9%	17	58.8%	17.6%

7	No additional product but adjustment of the patient's current cannabis THC:CBD ratio	11.8%	41.2%	5.9%	29.4%	0.0%	11.8%	17	52.9%	29.4%

The starting THC:CBD ratio for treating breakthrough pain in patients with chronic pain is:

1 #	Question	Strongly Disagree	Disagree	Neutral	Agree	Strongly agree	Abstain	Total	Strongly Disagree/ Disagree	Strongly Agree/ Agree
1	CBD predominant	17.7%	41.2%	23.5%	5.9%	5.9%	5.9%	17	58.8%	11.8%
2	Balanced THC:CBD	0.0%	5.9%	5.9%	64.7%	17.7%	5.9%	17	5.9%	82.4%
3	THC predominant	0.0%	17.7%	23.5%	29.4%	23.5%	5.9%	17	17.7%	52.9%
4	No additional product but adjustment of the patientâ€™s current cannabis THC:CBD ratio	23.5%	35.3%	5.9%	11.8%	5.9%	17.7%	17	58.8%	17.6%

The monitoring frequency of a patient who is STARTING on medical cannabis is:

1 #	Question	Strongly Disagree	Disagree	Neutral	Agree	Strongly agree	Abstain	Total	Strongly Disagree/ Disagree	Strongly Agree/ Agree
1	More frequently than once weekly	12.5%	50.0%	12.5%	18.8%	6.3%	0.0%	16	62.5%	25.0%
2	Once weekly	6.3%	37.5%	12.5%	25.0%	18.8%	0.0%	16	43.8%	43.8%
3	Twice monthly	0.0%	29.4%	11.8%	35.3%	17.7%	5.9%	17	29.4%	52.9%
4	Once monthly	11.8%	11.8%	11.8%	35.3%	23.5%	5.9%	17	23.5%	58.8%
5	Once every 2 months	37.5%	31.3%	6.3%	18.8%	0.0%	6.3%	16	68.8%	18.8%
6	> 2 months	73.3%	13.3%	6.7%	0.0%	0.0%	6.7%	15	86.7%	0.0%

The monitoring frequency of a patient who is STABLE on medical cannabis is:

1 #	Question	Strongly Disagree	Disagree	Neutral	Agree	Strongly agree	Abstain	Total	Strongly Disagree/ Disagree	Strongly Agree/ Agree
1	More frequently than once monthly	50.0%	25.0%	18.8%	6.3%	0.0%	0.0%	16	75.0%	6.3%
2	Monthly	18.8%	12.5%	18.8%	31.3%	12.5%	6.3%	16	31.3%	43.8%
3	Every 3 months	0.0%	18.8%	0.0%	43.8%	31.3%	6.3%	16	18.8%	75.0%
4	Every 6 months	6.3%	31.3%	18.8%	25.0%	12.5%	6.3%	16	37.5%	37.5%
5	> 6 months	31.3%	31.3%	12.5%	18.8%	0.0%	6.3%	16	62.5%	18.8%
6	Routine monitoring is not required	62.5%	31.3%	0.0%	0.0%	0.0%	6.3%	16	93.8%	0.0%

Medical cannabis should be stopped when:

1 #	Question	Strongly Disagree	Disagree	Neutral	Agree	Strongly agree	Abstain	Total	Strongly Disagree/ Disagree	Strongly Agree/ Agree
1	The patient has reached a > 30% reduction in pain intensity	35.3%	35.3%	11.8%	17.7%	0.0%	0.0%	17	70.6%	17.7%
2	The patient has reached an improvement in function	35.3%	35.3%	5.9%	17.7%	5.9%	0.0%	17	70.6%	23.5%
3	The patient experiences mild cannabis-related adverse effects	5.9%	64.7%	17.7%	11.8%	0.0%	0.0%	17	70.6%	11.8%
4	The patient experiences moderate or severe cannabis-related adverse effects	0.0%	0.0%	5.9%	52.9%	41.2%	0.0%	17	0.0%	94.1%
5	The medical cannabis is not providing pain relief after a dose increase	5.9%	29.4%	41.2%	17.7%	5.9%	0.0%	17	35.3%	23.5%
6	When the maximum agreed upon dose in mg is reached and not benefit	0.0%	5.9%	11.8%	52.9%	29.4%	0.0%	17	5.9%	82.4%
7	If a patient experiences loss of efficacy after a dosage increase	5.9%	35.3%	29.4%	17.7%	11.8%	0.0%	17	41.2%	29.4%
8	If the patient is not responded on two trials of cannabis varieties	11.8%	41.2%	29.4%	11.8%	5.9%	0.0%	17	52.9%	17.6%
9	If the patient has misuse or diversion	0.0%	5.9%	11.8%	29.4%	47.1%	5.9%	17	5.9%	76.5%
10	If the patient has legal troubles or motor vehicle accidents	0.0%	5.9%	35.3%	11.8%	41.2%	5.9%	17	5.9%	52.9%

CBD is associated with the following adverse effects:

1 #	Question	Strongly Disagree	Disagree	Neutral	Agree	Strongly agree	Abstain	Total	Strongly Disagree/ Disagree	Strongly Agree/ Agree
1	Anxiety	17.7%	47.1%	17.7%	11.8%	0.0%	5.9%	17	64.7%	11.8%
2	Dizziness	5.9%	41.2%	5.9%	41.2%	0.0%	5.9%	17	47.1%	41.2%
3	Fatigue or somnolence	5.9%	5.9%	11.8%	64.7%	5.9%	5.9%	17	11.8%	70.6%
4	Increase risk of falls	6.3%	43.8%	18.8%	25.0%	0.0%	6.3%	16	50.0%	25.0%
5	Reduced blood pressure	5.9%	52.9%	23.5%	11.8%	0.0%	5.9%	17	58.8%	11.8%
6	Diarrhea	0.0%	11.8%	29.4%	52.9%	0.0%	5.9%	17	11.8%	52.9%
7	Appetite loss	5.9%	47.1%	5.9%	35.3%	0.0%	5.9%	17	52.9%	35.3%
8	Acute Psychosis	35.3%	41.2%	5.9%	11.8%	0.0%	5.9%	17	76.5%	11.8%
9	Short-term memory loss	17.7%	52.9%	11.8%	11.8%	0.0%	5.9%	17	70.6%	11.8%

10	Tachycardia	23.5%	35.3%	5.9%	29.4%	0.0%	5.9%	17	58.8%	29.4%
11	Nausea and vomiting	11.8%	35.3%	11.8%	29.4%	5.9%	5.9%	17	47.1%	35.3%
12	Sleep Disturbances	11.8%	23.5%	17.7%	41.2%	0.0%	5.9%	17	35.3%	41.2%
13	Restlessness	17.7%	29.4%	11.8%	35.3%	0.0%	5.9%	17	47.1%	35.3%
14	Sensitivity reactions (e.g. rash, itch)	5.9%	47.1%	17.7%	23.5%	0.0%	5.9%	17	52.9%	23.5%

THC is associated with the following adverse effects:

	#	Question	Strongly Disagree	Disagree	Neutral	Agree	Strongly agree	Abstain	Total	Strongly Disagree/ Disagree	Strongly Agree/ Agree
1											
	1	Anxiety	0.0%	0.0%	17.7%	52.9%	29.4%	0.0%	17	0.0%	82.4%
	2	Dizziness	0.0%	0.0%	0.0%	58.8%	41.2%	0.0%	17	0.0%	100.0%
	3	Fatigue or somnolence	0.0%	0.0%	0.0%	58.8%	41.2%	0.0%	17	0.0%	100.0%
	4	Increase risk of falls	0.0%	0.0%	11.8%	52.9%	35.3%	0.0%	17	0.0%	88.2%
	5	Reduced blood pressure	0.0%	0.0%	17.7%	47.1%	29.4%	5.9%	17	0.0%	76.5%
	6	Diarrhea	5.9%	17.7%	29.4%	41.2%	5.9%	0.0%	17	23.5%	47.1%
	7	Appetite loss	17.7%	41.2%	11.8%	17.7%	5.9%	5.9%	17	58.8%	23.5%
	8	Acute Psychosis	5.9%	0.0%	0.0%	58.8%	35.3%	0.0%	17	5.9%	94.1%
	9	Short-term memory loss	0.0%	0.0%	5.9%	58.8%	35.3%	0.0%	17	0.0%	94.1%
	10	Tachycardia	0.0%	0.0%	5.9%	58.8%	35.3%	0.0%	17	0.0%	94.1%
	11	Nausea and vomiting	0.0%	5.9%	17.7%	41.2%	29.4%	5.9%	17	5.9%	70.6%
	12	Xerostomia	0.0%	11.8%	29.4%	17.7%	35.3%	5.9%	17	11.8%	52.9%
	13	Sensitivity reactions (e.g. rash, itch)	0.0%	23.5%	47.1%	17.7%	5.9%	5.9%	17	23.5%	23.5%

Goals for using medical cannabis include:

	#	Question	Strongly Disagree	Disagree	Neutral	Agree	Strongly agree	Abstain	Total	Strongly Disagree/ Disagree	Strongly Agree/ Agree
1	1	Improve overall analgesic efficacy	0.0%	0.0%	0.0%	35.3%	64.7%	0.0%	17	0.0%	100.0%
	2	Address breakthrough pain symptoms	0.0%	5.9%	17.7%	47.1%	29.4%	0.0%	17	5.9%	76.5%
	3	Address episodic symptoms and exacerbations	0.0%	5.9%	17.7%	41.2%	35.3%	0.0%	17	5.9%	76.5%
	4	Improvement in disease specific symptoms and symptom burden	0.0%	0.0%	0.0%	47.1%	52.9%	0.0%	17	0.0%	100.0%
	5	Spare opioids and support opioid tapers	0.0%	0.0%	0.0%	35.3%	64.7%	0.0%	17	0.0%	100.0%
	6	Reduce benzodiazepine use	0.0%	0.0%	5.9%	35.3%	58.8%	0.0%	17	0.0%	94.1%
	7	Reduce skeletal muscle relaxant use	0.0%	5.9%	5.9%	58.8%	29.4%	0.0%	17	5.9%	88.2%
	8	Reduce hypnotic use	0.0%	0.0%	17.7%	47.1%	35.3%	0.0%	17	0.0%	82.4%
	9	Reduce NSAID use	0.0%	11.8%	17.7%	52.9%	17.7%	0.0%	17	11.8%	70.6%
	10	Reduce antidepressant use	0.0%	29.4%	23.5%	29.4%	17.7%	0.0%	17	29.4%	47.1%
	11	Reduce neuroleptic use	0.0%	11.8%	29.4%	35.3%	23.5%	0.0%	17	11.8%	58.8%
	12	Reduce corticosteroid use	5.9%	17.7%	35.3%	17.7%	23.5%	0.0%	17	23.5%	41.2%
	13	Reduce legal substance use (e.g. alcohol, tobacco)	0.0%	17.7%	23.5%	35.3%	23.5%	0.0%	17	17.7%	58.8%
	14	Reduce illicit substance use	0.0%	11.8%	11.8%	41.2%	35.3%	0.0%	17	11.8%	76.5%
	15	Mitigate opioid-related adverse effects	0.0%	0.0%	0.0%	52.9%	47.1%	0.0%	17	0.0%	100.0%
	16	Reduce opioid withdrawal symptom	0.0%	5.9%	17.7%	35.3%	41.2%	0.0%	17	5.9%	76.5%
	17	Improve quality of life	0.0%	0.0%	0.0%	29.4%	70.6%	0.0%	17	0.0%	100.0%
	18	Improve function	0.0%	0.0%	0.0%	17.7%	82.4%	0.0%	17	0.0%	100.0%
	19	Improve self-efficacy	0.0%	0.0%	0.0%	35.3%	58.8%	5.9%	17	0.0%	94.1%
	20	Improve sleep	0.0%	0.0%	0.0%	17.7%	82.4%	0.0%	17	0.0%	100.0%
	21	Improve mood	0.0%	0.0%	5.9%	29.4%	64.7%	0.0%	17	0.0%	94.1%
	22	Reduce anhedonia	0.0%	0.0%	11.8%	52.9%	35.3%	0.0%	17	0.0%	88.2%
	23	Reduce anxiety	0.0%	0.0%	11.8%	47.1%	41.2%	0.0%	17	0.0%	88.2%

The place in therapy for medical cannabis for Chronic inflammatory pain is:

	#	Question	Strongly Disagree	Disagree	Neutral	Agree	Strongly agree	Abstain	Total	Strongly Disagree/ Disagree	Strongly Agree/ Agree
	1	As a first-line therapy after non-pharmacotherapy options	29.4%	64.7%	0.0%	5.9%	0.0%	0.0%	17	94.1%	5.9%
1	2	Before acetaminophen (paracetamol) and NSAIDs	29.4%	58.8%	5.9%	5.9%	0.0%	0.0%	17	88.2%	5.9%
	3	Before antidepressants	5.9%	58.8%	11.8%	5.9%	17.7%	0.0%	17	64.7%	23.5%
	4	Before anti-epileptics	5.9%	52.9%	11.8%	11.8%	17.7%	0.0%	17	58.8%	29.4%
	5	Before opioids	5.9%	29.4%	5.9%	23.5%	35.3%	0.0%	17	35.3%	58.8%

The place in therapy for medical cannabis for Chronic nociceptive pain with supraspinal sensitisation is:

	#	Question	Strongly Disagree	Disagree	Neutral	Agree	Strongly agree	Abstain	Total	Strongly Disagree/ Disagree	Strongly Agree/ Agree
1											
	1	As a first-line therapy after non-pharmacotherapy options	17.7%	58.8%	5.9%	5.9%	11.8%	0.0%	17	76.5%	17.6%
	2	Before acetaminophen (paracetamol) and NSAIDs	23.5%	47.1%	17.7%	0.0%	11.8%	0.0%	17	70.6%	11.8%
	3	Before antidepressants	0.0%	58.8%	23.5%	11.8%	5.9%	0.0%	17	58.8%	17.6%
	4	Before anti-epileptics	0.0%	41.2%	35.3%	17.7%	5.9%	0.0%	17	41.2%	23.5%
	5	Before opioids	5.9%	11.8%	5.9%	41.2%	35.3%	0.0%	17	17.6%	76.5%

The place in therapy for medical cannabis for Chronic nociceptive pain without supraspinal sensitisation is:

	#	Question	Strongly Disagree	Disagree	Neutral	Agree	Strongly agree	Abstain	Total	Strongly Disagree/ Disagree	Strongly Agree/ Agree
1											
	1	As a first-line therapy after non-pharmacotherapy options	41.2%	52.9%	0.0%	0.0%	5.9%	0.0%	17	94.1%	5.9%
	2	Before acetaminophen (paracetamol) and NSAIDs	29.4%	58.8%	5.9%	0.0%	5.9%	0.0%	17	88.2%	5.9%
	3	Before antidepressants	11.8%	52.9%	23.5%	5.9%	5.9%	0.0%	17	64.7%	11.8%
	4	Before anti-epileptics	11.8%	47.1%	23.5%	11.8%	5.9%	0.0%	17	58.8%	17.6%
	5	Before opioids	5.9%	23.5%	5.9%	29.4%	35.3%	0.0%	17	29.4%	64.7%

The place in therapy for medical cannabis for Chronic neuropathic pain is:

	#	Question	Strongly Disagree	Disagree	Neutral	Agree	Strongly agree	Abstain	Total	Strongly Disagree/ Disagree	Strongly Agree/ Agree
1											
	1	As a first-line therapy after non-pharmacotherapy options	11.8%	64.7%	11.8%	0.0%	11.8%	0.0%	17	76.5%	11.8%
	2	Before acetaminophen (paracetamol) and NSAIDs	23.5%	23.5%	17.7%	23.5%	11.8%	0.0%	17	47.1%	35.3%
	3	Before antidepressants	5.9%	52.9%	17.7%	17.7%	5.9%	0.0%	17	58.8%	23.5%
	4	Before anti-epileptics	5.9%	47.1%	23.5%	17.7%	5.9%	0.0%	17	52.9%	23.5%
	5	Before opioids	0.0%	17.7%	0.0%	35.3%	47.1%	0.0%	17	17.7%	82.4%

The place in therapy for medical cannabis for Chronic visceral pain is:

	#	Question	Strongly Disagree	Disagree	Neutral	Agree	Strongly agree	Abstain	Total	Strongly Disagree/ Disagree	Strongly Agree/ Agree
1											
	1	As a first-line therapy after non-pharmacotherapy options	23.5%	52.9%	11.8%	0.0%	11.8%	0.0%	17	76.5%	11.8%
	2	Before acetaminophen (paracetamol) and NSAIDs	11.8%	41.2%	17.7%	17.7%	11.8%	0.0%	17	52.9%	29.4%
	3	Before antidepressants	5.9%	47.1%	17.7%	17.7%	11.8%	0.0%	17	52.9%	29.4%
	4	Before anti-epileptics	5.9%	35.3%	23.5%	23.5%	11.8%	0.0%	17	41.2%	35.3%
	5	Before opioids	0.0%	17.7%	5.9%	35.3%	41.2%	0.0%	17	17.7%	76.5%

The place in therapy for medical cannabis for Chronic traumatic pain is:

	#	Question	Strongly Disagree	Disagree	Neutral	Agree	Strongly agree	Abstain	Total	Strongly Disagree/ Disagree	Strongly Agree/ Agree
1											
	1	As a first-line therapy after non-pharmacotherapy options	23.5%	58.8%	5.9%	0.0%	5.9%	5.9%	17	82.4%	5.9%
	2	Before acetaminophen (paracetamol) and NSAIDs	11.8%	64.7%	11.8%	0.0%	5.9%	5.9%	17	76.5%	5.9%
	3	Before antidepressants	5.9%	47.1%	23.5%	11.8%	5.9%	5.9%	17	52.9%	17.6%
	4	Before anti-epileptics	5.9%	35.3%	35.3%	5.9%	11.8%	5.9%	17	41.2%	17.6%
	5	Before opioids	5.9%	17.7%	5.9%	29.4%	35.3%	5.9%	17	23.5%	64.7%

The place in therapy for medical cannabis for Chronic cancer pain is:

	#	Question	Strongly Disagree	Disagree	Neutral	Agree	Strongly agree	Abstain	Total	Strongly Disagree/ Disagree	Strongly Agree/ Agree
1											
	1	As a first-line therapy after non-pharmacotherapy options	23.5%	47.1%	5.9%	5.9%	11.8%	5.9%	17	70.6%	17.6%
	2	Before acetaminophen (paracetamol) and NSAIDs	23.5%	41.2%	11.8%	5.9%	11.8%	5.9%	17	64.7%	17.6%
	3	Before antidepressants	5.9%	35.3%	17.7%	23.5%	11.8%	5.9%	17	41.2%	35.3%
	4	Before anti-epileptics	5.9%	29.4%	23.5%	23.5%	11.8%	5.9%	17	35.3%	35.3%
	5	Before opioids	6.3%	25.0%	12.5%	31.3%	18.8%	6.3%	16	31.3%	50.0%

The place in therapy for medical cannabis for Palliative pain is:

1

#	Question	Strongly Disagree	Disagree	Neutral	Agree	Strongly agree	Abstain	Total	Strongly Disagree/ Disagree	Strongly Agree/ Agree
1	As a first-line therapy after non-pharmacotherapy options	23.5%	35.3%	5.9%	17.7%	17.7%	0.0%	17	58.8%	35.3%
2	Before acetaminophen (paracetamol) and NSAIDs	23.5%	29.4%	11.8%	17.7%	17.7%	0.0%	17	52.9%	35.3%
3	Before antidepressants	11.8%	29.4%	17.7%	23.5%	17.7%	0.0%	17	41.2%	41.2%
4	Before anti-epileptics	11.8%	23.5%	17.7%	29.4%	17.7%	0.0%	17	35.3%	47.1%
5	Before opioids	11.8%	17.7%	23.5%	29.4%	17.7%	0.0%	17	29.4%	47.1%
