# Supplementary material for: Consensus recommendations on dosing and administration of medical cannabis to treat chronic pain: results of a modified Delphi process
Source: J Cannabis Res. 2021 Jul 2;3:22. doi: 10.1186/s42238-021-00073-1 (PMC8252988; doi:10.1186/s42238-021-00073-1)
Supplement: Supplementary file 3 — Additional file 3. Second pre vote results. [file 42238_2021_73_MOESM3_ESM.pdf]

Consensus Voting Round 2 Results

Consensus Reached with ≥75%

n=17

Rationale for using medical cannabis in patientswith chronic pain, include:

1

#	Question	Strongly Disagree	Disagree	Neutral	Agree	Strongly agree	Abstain	Total	Strongly Disagree/ Disagree	Strongly Agree/ Agree
1	Reduction in anti-depressant use	0%	35%	0%	47%	18%	0%	17	35%	65%
2	Reduction in anti-epileptic use	0%	29%	12%	35%	24%	0%	17	29%	59%
3	Patient preference for cannabis use	6%	12%	18%	53%	12%	0%	17	18%	65%

Medical cannabis is likely effective for

1

#	Question	Strongly Disagree	Disagree	Neutral	Agree	Strongly agree	Abstain	Total	Strongly Disagre	Strongly Agree
1	Mixed pain	0%	0%	6%	82%	12%	0%	17	0%	94%
2	Neuropathic pain	0%	0%	0%	35%	65%	0%	17	0%	100%
3	Inflammatory pain	0%	0%	12%	82%	6%	0%	17	0%	88%
4	Nociplastic pain	0%	0%	18%	65%	12%	6%	17	0%	77%
5	Chronic headache/migraines	0%	0%	41%	47%	12%	0%	17	0%	59%

The minimum age for use of CBD in patients with chronic pain is:

1

#	Answer	%
1	25 years of age	6%
2	21 years of age	6%
3	18 years of age	12%
4	No minimum age	76%
5	Total	17

Medical cannabis should be avoided in patients with chronic pain with the following conditions:

1

#	Question	Strongly Disagree	Disagree	Neutral	Agree	Strongly agree	Abstain	Total	Strongly Disagree/ Disagree	Strongly Agree/ Agree
1	A patient with mood disorder	18%	53%	24%	6%	0%	0%	17	71%	6%
2	A patient who is taking high doses benzodiazepines or other sedating medications prescribed or over the counter	12%	53%	12%	24%	0%	0%	17	65%	24%
3	A patient with stable cardiovascular disease	12%	82%	6%	0%	0%	0%	17	94%	0%
4	A patient with unstable cardiovascular disease	0%	18%	18%	47%	18%	0%	17	18%	65%
5	A patient with severe renal disease	18%	47%	24%	12%	0%	0%	17	65%	12%
6	A patient with immunosuppression	29%	35%	29%	0%	0%	6%	17	64%	0%

Medical cannabis should be avoided in patients with chronic pain being administered the following medications:

1

#	Question	Strongly Disagree	Disagree	Neutral	Agree	Strongly agree	Abstain	Total	Strongly Disagree/ Disagree	Strongly Agree/ Agree
1	Warfarin	12%	53%	12%	24%	0%	0%	17	65%	24%
2	Direct oral anticoagulants (DOACs)	12%	53%	12%	6%	0%	0%	17	65%	18%
3	Anti-psychotics	18%	41%	18%	24%	0%	0%	17	59%	24%
4	Immunotherapy agents for oncology (e.g. checkpoint inhibitors)	24%	24%	24%	24%	6%	0%	17	48%	30%

The initiating THC:CBD ratio for patients with chronic pain requiring routine dosing and titration should be:

1

#	Question	Strongly Disagree	Disagree	Neutral	Agree	Strongly agree	Abstain	Total	Strongly Disagree/ Disagree	Strongly Agree/ Agree
1	CBD predominant	6%	12%	12%	29%	41%	0%	17	18%	70%
2	CBD predominant during the day and add THC predominant at bedtime	6%	24%	24%	35%	12%	0%	17	30%	47%
3	Balanced THC:CBD	0%	24%	12%	47%	18%	0%	17	24%	65%
4	THC predominant	24%	41%	6%	24%	6%	0%	17	65%	30%

The initiating CBD daily dose for routine dosing and titration is: (total amount in 24 hours)

1

#	Answer	%
1	5 mg	12%
2	10 mg	71%
3	20 mg	6%
4	50 mg	12%
5	> 50 mg	0%
6	Total	17

For the routine arm, the recommended initiating dosing frequency for CBD is: (Daily dose divided into this frequency)

1

#	Answer	%
1	Once daily	24%
2	Twice daily	76%
3	Total	17

The recommended CBD titration amount for routine dosing and titration is:

1

#	Answer	%
1	5 mg	29%
2	10 mg	47%
3	20 mg	24%
4	Total	17

The recommended CBD up-titration frequency for routine dosing and adminstration is:

1

#	Answer	%
1	Daily	13%
2	Every other day	31%
3	Once a week	56%
4	Total	16

For routine dosing and titration, after initiating with a CBD predominant variety and target symptom relief has not been obtained, THC should be introduced:

1

#	Question	Strongly Disagree	Disagree	Neutral	Agree	Strongly agree	Abstain	Total	Strongly Disagree/ Disagree	Strongly Agree/ Agree
1	At bedtime once patient is taking > 20mg of CBD	12%	12%	47%	24%	6%	0%	17	24%	30%
2	At bedtime once patient is taking > 40mg of CBD	12%	12%	18%	35%	24%	0%	17	24%	59%
3	At bedtime when the patient is on maximally tolerated CBD dose	0%	24%	24%	24%	29%	0%	17	24%	53%
4	At bedtime when the patient is on maximal affordable CBD dose	0%	18%	18%	35%	24%	6%	17	18%	59%
5	I do not recommend initiating with CBD predominant before initiating THC	24%	29%	12%	12%	18%	6%	17	53%	30%

The recommended initiating THC daily dose for routine dosing and titration is: (amount for 24 hours)

1

#	Answer	%
1	1 mg	13%
2	2.5 mg	88%
3	Total	16

The recommended THC titration amount for routine dosing and titration is:

1

#	Answer	%
1	1 mg	12%
2	2.5 mg	88%
3	Total	17

The recommended THC up-titration frequency for routine dosing and titration is:

1

#	Answer	%
1	Daily	0%
2	Every other day	47%
3	Once a week	41%

4	Every 2 weeks	6%
5	Abstain	6%
6	Total	17

The recommended maximum daily dose of CBD for routine dosing and titration is:

1

#	Answer	%
1	50 mg	0%
2	100 mg	29%
3	300 mg	29%
4	600 mg	0%
5	No maximum daily dose	41%
6	Abstain	0%
	Total	17

The recommended maximum daily dose of THC for routine dosing and titration is:

1

#	Answer	%
1	10 mg	0%
2	20 mg	29%
3	40 mg	47%
4	80 mg	6%
5	No maximum daily dose	18%
6	Total	17

The patients and conditions that should be considered for rapid medical cannabis dosing and titration are:

1

#	Question	Strongly Disagree	Disagree	Neutral	Agree	Strongly agree	Abstain	Total	Strongly Disagree/ Disagree	Strongly Agree/ Agree
1	The patient is taking > 90 mg morphine equivalent dose (MED)	6%	18%	29%	35%	12%	0%	17	24%	47%
2	The patient who is currently using opioids, gabapentinoids or SNRIs	0%	41%	41%	12%	6%	0%	17	41%	18%
3	The patient who has been unsuccessfully treated with opioids, gabapentinoids or SNRIs	0%	29%	29%	35%	6%	0%	17	29%	41%
4	The patient is experiencing primarily neuropathic pain	0%	29%	24%	41%	6%	0%	17	29%	47%
5	Lack of efficacy at low/moderate cannabis doses	0%	24%	29%	24%	24%	0%	17	24%	48%

The recommended initiating THC:CBD ratio for rapid dosing and titration is:

1

#	Question	Strongly Disagree	Disagree	Neutral	Agree	Strongly agree	Abstain	Total	Strongly Disagree/ Disagree	Strongly Agree/ Agree
1	CBD predominant in the day and THC predominant at bedtime	0%	18%	41%	35%	6%	0%	17	18%	41%
2	Balanced THC:CBD	0%	0%	12%	59%	29%	0%	17	0%	88%
3	THC predominant	6%	24%	35%	29%	6%	0%	17	30%	35%

The recommended initiating THC daily dose for rapid dosing and titration is: (amount for 24 hours)

1

#	Answer	%
1	2.5 mg	35%
2	5 mg	53%
3	10 mg	12%
4	Abstain	0%
5	Total	17

The recommended initiating dosing frequency of THC for rapid dosing and titration is: (Daily dose divided into this frequency)

1

#	Answer	%
1	Once daily	35%
2	Twice daily	53%
3	Three times daily	12%
4	Abstain	0%
5	Total	17

The recommended THC titration amount for rapid dosing and titration is:

1

#	Answer	%
1	1 mg	6%
2	5 mg	29%
3	10 mg	0%
4	2.5 mg	65%
5	Abstain	0%
6	Total	17

The recommended THC up-titration frequency for rapid dosing and titration is:

1

#	Answer	%
1	Daily	12%
2	Every other day	82%
3	Once a week	6%
4	Abstain	0%
5	Total	17

The recommended maximum daily dose of THC for rapid dosing and titration is:

1

#	Answer	%
1	20 mg	6%
2	40 mg	65%
3	80 mg	18%
4	No maximum daily dose	12%
5	Abstain	0%
6	Total	17

The recommended initiating CBD daily dose for rapid dosing and titration is: (total amount in 24 hours)

1

#	Answer	%
1	5 mg	12%
2	10 mg	24%
3	20 mg	24%
4	> 50 mg	12%
5	I would initiate with THC only	12%
6	2.5 mg	0%
7	I would initiate with balanced THC:CBD	18%
8	Abstain	0%
9	Total	17

The recommended starting dosing frequency of CBD for rapid dosing and titration is: (Daily dose divided into this frequency)

1

#	Answer	%
1	Once daily	0%
2	Twice daily	71%
3	Three times daily	18%
4	I would start with THC only	0%
5	Abstain	0%
6	I would initiate with balanced THC:CBD	12%
7	Total	17

The recommended CBD titration amount for rapid dosing and titration:

1

#	Answer	%
1	5 mg	12%
2	10 mg	41%
3	20 mg	12%
4	> 20 mg	18%
5	I would start with THC only	6%
6	Abstain	0%
7	2.5 mg	0%

8	I would initiate with balanced THC:CBD	12%
9	Total	1700%

The recommended CBD up-titration frequency for rapid dosing and titration:

1

#	Answer	%
1	Daily	12%
2	Every other day	65%
3	Once a week	6%
4	Abstain	0%
5	I would initiate with THC only	6%
6	I would initiate with balanced THC:CBD	12%
7	Total	17

The recommended maximum daily dose of CBD for rapid dosing and titration:

1

#	Answer	%
1	100 mg	24%
2	300 mg	18%
3	600 mg	0%
4	No maximum daily dose	29%
5	I would start with THC only	12%
6	I would initiate with balanced THC:CBD	18%
7	Total	17

The recommended initiating THC:CBD ratio for patients with chronic pain requiring conservative dosing and titration is:

1

#	Question	Strongly Disagree	Disagree	Neutral	Agree	Strongly agree	Abstain	Total	Strongly Disagree/ Disagree	Strongly Agree/ Agree
1	CBD predominant	6%	0%	12%	29%	53%	0%	17	6%	82%
2	CBD predominant in the day and THC predominant at bedtime	0%	20%	33%	33%	13%	0%	15	20%	46%
3	Balanced THC:CBD	7%	20%	20%	47%	7%	0%	15	27%	54%

The initiating CBD daily dose for patients with chronic pain requiring conservative dosing and titration: (total amount in 24 hours)

1

#	Answer	%
1	5 mg	41%
2	10 mg	35%
3	20 mg	24%
4	Total	17

The recommended CBD titration amount for conservative dosing and titration is:

1

#	Answer	%
1	1 mg	6%
2	5 mg	53%
3	10 mg	24%
4	20 mg	6%
5	> 20 mg	12%
6	Total	17

The recommended CBD up-titration frequency for conservative dosing and titration is:

1

#	Answer	%
1	Daily	6%
2	Every other day	24%
3	Once a week	53%
4	Every 2 weeks	18%
5	Total	17

The recommended maximum daily dose of CBD for conservative dosing and titration is:

1

#	Answer	%
1	50 mg	6%
2	100 mg	35%
3	300 mg	29%
4	No maximum daily dose	29%
5	Abstain	0%
6	Total	17

For conservative dosing, after initiating with a CBD predominant variety and target symptom relief has not been obtained, THC should be introduced:

1

#	Question	Strongly Disagree	Disagree	Neutral	Agree	Strongly agree	Abstain	Total	Strongly Disagree/ Disagree	Strongly Agree/ Agree
1	At bedtime once patient is taking > 20mg of CBD	6%	19%	50%	6%	13%	6%	16	25%	19%
2	At bedtime once patient is taking > 40mg of CBD	6%	12%	35%	35%	12%	0%	17	18%	47%
3	At bedtime when the patient is on maximally tolerated CBD dose	0%	24%	12%	29%	35%	0%	17	24%	64%
4	At bedtime when the patient is on maximal affordable CBD dose	0%	13%	13%	25%	44%	6%	16	13%	69%
5	I do not recommend starting with CBD predominant before initiating THC	31%	25%	19%	13%	13%	0%	16	56%	26%

The recommended THC up-titration frequency for patients with chronic pain requiring conservative dosing and titration:

1

#	Answer	%
1	Every other day	18%
2	Once a week	59%
3	Every 2 weeks	24%
4	Total	17

The recommended maximum daily dose of THC for patients with chronic pain requiring conservative dosing and titration:

1

#	Answer	%
1	10 mg	12%
2	20 mg	53%
3	40 mg	18%
4	80 mg	12%
5	No maximum daily dose	6%
6	Total	17

The preferred method for inhaled medical cannabis is:

1

#	Question	Strongly Disagree	Disagree	Neutral	Agree	Strongly agree	Abstain	Total	Strongly Disagree/ Disagree	Strongly Agree/ Agree
1	Smoking dried cannabis flower	59%	24%	6%	0%	0%	0%	12%	83%	0%
2	Vaporization of dried cannabis flower	0%	0%	6%	0%	41%	41%	12%	0%	41%
3	Vaporization of cannabis extract	6%	12%	12%	6%	24%	24%	18%	18%	30%
4	I will not prescribe/authorize inhaled medical cannabis for chronic pain management	59%	12%	0%	12%	6%	12%	0%	71%	18%

The patients where inhaled cannabis should be considered for chronic pain management.

1

#	Question	Strongly Disagree	Disagree	Neutral	Agree	Strongly agree	Abstain	Total	Strongly Disagree/ Disagree	Strongly Agree/ Agree
1	Patients currently using inhaled cannabis	0%	18%	18%	35%	18%	12%	17	18%	53%
2	Patients refusing to use oral cannabis extract	6%	12%	12%	41%	18%	12%	17	18%	59%
3	Abstain	15%	15%	23%	8%	0%	38%	13	30%	8%

Q51 - The recommended initiating THC:CBD ratio for patients with chronic pain for inhaled use:

1

#	Question	Strongly Disagree	Disagree	Neutral	Agree	Strongly agree	Abstain	Total	Strongly Disagree/ Disagree	Strongly Agree/ Agree
1	CBD predominant	6%	18%	35%	6%	24%	12%	17	24%	30%
2	CBD predominant in the day and THC predominant at bedtime	0%	29%	12%	41%	6%	12%	17	29%	47%
3	Balanced THC:CBD	0%	6%	0%	53%	29%	12%	17	6%	82%
4	THC predominant	24%	24%	0%	29%	12%	12%	17	48%	41%

The starting dose of medical cannabis for inhaled use:

	#	Answer	%
1	#	Answer	%
	1	1 inhalation	59%
	2	2 inhalations	24%
	3	Abstain	18%
	4	Total	17

The recommended daily dosing for inhaled cannabis is:

	#	Answer	%
1	#	Answer	%
	1	Twice daily when required	24%
	2	Four times daily when required	24%
	3	Every 4 hours when required	35%
	4	I don't recommend inhaled cannabis	0%
	5	Abstain	18%
	6	Total	17

The recommended up-titration amount for medical cannabis for inhaled use is:

	#	Answer	%
1	#	Answer	%
	1	1 inhalation	71%
	2	2 inhalations	12%
	3	I don't recommend inhaled cannabis	0%
	4	Abstain	18%
	5	Total	17

The THC:CBD ratio for treating breakthrough pain in patients with chronic pain is:

	#	Answer	Strongly Disagree	Disagree	Neutral	Agree	Strongly agree	Abstain	Total	Strongly Disagree/ Disagree	Strongly Agree/ Agree
1	#	Answer	Strongly Disagree	Disagree	Neutral	Agree	Strongly agree	Abstain	Total	Strongly Disagree/ Disagree	Strongly Agree/ Agree
	1	Balanced THC:CBD	0%	6%	12%	53%	29%	6%	17	6%	82%
	2	THC predominant	0%	19%	6%	50%	25%	0%	17	19%	75%

The recommended dosing and administration considerations for treating breakthrough pain with medical cannabis are:

	#	Question	Strongly Disagree	Disagree	Neutral	Agree	Strongly agree	Abstain	Total	Strongly Disagree/ Disagree	Strongly Agree/ Agree
1	1	Clinicians should increase the dose of the same cannabis product to manage breakthrough pain	0%	18%	12%	47%	18%	6%	17	18%	65%
	2	Clinicians should increase the THC dose to manage the patient‚Äôs breakthrough pain	0%	18%	24%	41%	18%	0%	17	18%	59%
	3	Clinicians should add as needed oral cannabis to manage breakthrough pain	18%	12%	12%	41%	18%	0%	17	30%	59%
	4	Clinicians should increase the CBD dose to manage the patient‚Äôs breakthrough pain	12%	47%	24%	18%	0%	0%	17	59%	18%

The monitoring frequency of a patient who is STARTING on medical cannabis is:

	#	Question	Strongly Disagree	Disagree	Neutral	Agree	Strongly agree	Abstain	Total	Strongly Disagree/ Disagree	Strongly Agree/ Agree
1	#	Question	Strongly Disagree	Disagree	Neutral	Agree	Strongly agree	Abstain	Total	Strongly Disagree/ Disagree	Strongly Agree/ Agree
	1	Once weekly	13%	19%	31%	25%	13%	0%	16	32%	38%
	2	Twice monthly	0%	24%	6%	47%	24%	0%	17	24%	71%
	3	Once monthly	6%	19%	19%	19%	38%	0%	16	25%	57%

Medical cannabis titration should be stopped when:

	#	Question	Strongly Disagree	Disagree	Neutral	Agree	Strongly agree	Abstain	Total	Strongly Disagree/ Disagree	Strongly Agree/ Agree
1	#	Question	Strongly Disagree	Disagree	Neutral	Agree	Strongly agree	Abstain	Total	Strongly Disagree/ Disagree	Strongly Agree/ Agree
	1	The patient has reached a > 30% reduction in pain intensity	12%	18%	6%	59%	6%	0%	17	30%	65%
	2	The patient has reached an improvement in function	6%	0%	18%	47%	29%	0%	17	6%	76%
	3	The patient experiences mild cannabis-related adverse effects	0%	41%	12%	35%	12%	0%	17	41%	47%
	4	The patient's chronic pain goals are being met	0%	0%	0%	29%	71%	0%	17	0%	100%

Medical cannabis treatment should be stopped and discontinued when:

	#	Question	Strongly Disagree	Disagree	Neutral	Agree	Strongly agree	Abstain	Total	Strongly Disagree/ Disagree	Strongly Agree/ Agree
1	#	Question	Strongly Disagree	Disagree	Neutral	Agree	Strongly agree	Abstain	Total	Strongly Disagree/ Disagree	Strongly Agree/ Agree
	1	The patient experiences mild cannabis-related adverse effects	18%	65%	12%	6%	0%	0%	17	83%	6%
	2	The patient experiences moderate or severe cannabis-related adverse effects	0%	6%	0%	53%	41%	0%	17	6%	94%
	3	The medical cannabis is not providing pain relief after a dose increase	6%	29%	29%	29%	6%	0%	17	35%	35%
	4	The maximum agreed upon dose in mg is reached and does not benefit the patient	6%	0%	6%	53%	35%	0%	17	6%	88%
	5	A patient experiences loss of efficacy after several dosage increases (Tolerance develops)	0%	18%	18%	53%	12%	0%	17	18%	65%
	6	The patient has not responded on two different cannabis varieties	18%	41%	12%	24%	6%	0%	17	59%	30%
	7	The patient has misuse or diversion	0%	12%	6%	29%	53%	0%	17	12%	82%
	8	The patient has legal troubles or motor vehicle accidents	0%	12%	12%	24%	47%	6%	17	12%	71%

CBD is associated with the following adverse effects:

	#	Question	Strongly Disagree	Disagree	Neutral	Agree	Strongly agree	Abstain	Total	Strongly Disagree/ Disagree	Strongly Agree/ Agree
1	#	Question	Strongly Disagree	Disagree	Neutral	Agree	Strongly agree	Abstain	Total	Strongly Disagree/ Disagree	Strongly Agree/ Agree
	1	Anxiety	29%	35%	18%	12%	6%	0%	17	64%	18%
	2	Fatigue or somnolence	6%	6%	24%	53%	12%	0%	17	12%	65%
	3	Short-term memory loss	24%	41%	18%	12%	6%	0%	17	65%	18%

THC is associated with the following adverse effects:

	#	Question	Strongly Disagree	Disagree	Neutral	Agree	Strongly agree	Abstain	Total	Strongly Disagree/ Disagree	Strongly Agree/ Agree
1	#	Question	Strongly Disagree	Disagree	Neutral	Agree	Strongly agree	Abstain	Total	Strongly Disagree/ Disagree	Strongly Agree/ Agree
	1	Diarrhea	6%	47%	6%	35%	0%	6%	17	53%	35%
	2	Appetite loss	12%	59%	6%	18%	0%	6%	17	71%	18%
	3	Xerostomia	0%	6%	24%	18%	47%	6%	17	6%	65%
	4	Sensitivity reactions (e.g. rash, itch)	0%	24%	53%	24%	0%	0%	17	24%	24%
	5	Nausea and vomiting	0%	18%	18%	59%	6%	0%	17	18%	65%

Goals for using medical cannabis include:

	#	Question	Strongly Disagree	Disagree	Neutral	Agree	Strongly agree	Abstain	Total	Strongly Disagree/ Disagree	Strongly Agree/ Agree
1	#	Question	Strongly Disagree	Disagree	Neutral	Agree	Strongly agree	Abstain	Total	Strongly Disagree/ Disagree	Strongly Agree/ Agree
	1	Reduce NSAID use	0%	0%	12%	71%	18%	0%	17	0%	89%
	2	Reduce antidepressant use	0%	29%	18%	41%	12%	0%	17	29%	53%
	3	Reduce neuroleptic use	0%	29%	18%	41%	12%	0%	17	29%	53%
	4	Reduce corticosteroid use	0%	12%	18%	47%	18%	6%	17	12%	65%
	5	Reduce legal substance use (e.g. alcohol, tobacco)	0%	12%	6%	53%	24%	6%	17	12%	77%

The place in therapy for medical cannabis for Mixed pain is:

	#	Question	Strongly Disagree	Disagree	Neutral	Agree	Strongly agree	Abstain	Total	Strongly Disagree/ Disagree	Strongly Agree/ Agree
1	#	Question	Strongly Disagree	Disagree	Neutral	Agree	Strongly agree	Abstain	Total	Strongly Disagree/ Disagree	Strongly Agree/ Agree
	1	As a first-line therapy after non-pharmacotherapy options	12%	82%	0%	6%	0%	0%	17	94%	6%
	2	Before acetaminophen (paracetamol) and NSAIDs	12%	76%	6%	0%	6%	0%	17	88%	6%
	3	Before antidepressants without signs of major depressive disorder	0%	71%	6%	24%	0%	0%	17	71%	24%
	4	Before anti-epileptics	0%	53%	18%	18%	12%	0%	17	53%	30%
	5	Before opioids	0%	6%	18%	47%	29%	0%	17	6%	76%

The place in therapy for medical cannabis for Neuropathic pain is:

	#	Question	Strongly Disagree	Disagree	Neutral	Agree	Strongly agree	Abstain	Total	Strongly Disagree/ Disagree	Strongly Agree/ Agree
1	1	As a first-line therapy after non-pharmacotherapy options	0%	88%	0%	6%	6%	0%	17	88%	12%

2	Before acetaminophen (paracetamol) and NSAIDs	6%	59%	0%	24%	12%	0%	17	65%	36%
3	Before antidepressants without signs of major depressive disorder	0%	76%	0%	18%	6%	0%	17	76%	24%
4	Before anti-epileptics	0%	76%	6%	6%	12%	0%	17	76%	18%
5	Before opioids	0%	0%	24%	35%	41%	0%	17	0%	76%

The place in therapy for medical cannabis for Inflammatory pain is:

#	Question	Strongly Disagree	Disagree	Neutral	Agree	Strongly agree	Abstain	Total	Strongly Disagree/ Disagree	Strongly Agree/ Agree
1	As a first-line therapy after non-pharmacotherapy options	12%	71%	12%	6%	0%	0%	17	83%	6%
2	Before acetaminophen (paracetamol) and NSAIDs	29%	71%	0%	0%	0%	0%	17	100%	0%
3	Before antidepressants without signs of major depressive disorder	6%	41%	6%	35%	12%	0%	17	47%	47%
4	Before anti-epileptics	6%	18%	12%	47%	18%	0%	17	24%	65%
5	Before opioids	6%	6%	18%	47%	24%	0%	17	12%	71%

1

The place in therapy for medical cannabis for Nociplastic pain is:

1

#	Question	Strongly Disagree	Disagree	Neutral	Agree	Strongly agree	Abstain	Total	Strongly Disagree/ Disagree	Strongly Agree/ Agree
1	As a first-line therapy after non-pharmacotherapy options	12%	65%	6%	12%	0%	6%	17	77%	12%
2	Before acetaminophen (paracetamol) and NSAIDs	18%	71%	0%	0%	6%	6%	17	89%	6%
3	Before antidepressants without signs of major depressive disorder	12%	53%	6%	18%	6%	6%	17	65%	24%
4	Before anti-epileptics	12%	53%	12%	6%	12%	6%	17	65%	18%
5	Before opioids	6%	6%	6%	53%	24%	6%	17	12%	77%

The place in therapy for medical cannabis for Chronic Headache/Migraine is:

1

#	Question	Strongly Disagree	Disagree	Neutral	Agree	Strongly agree	Abstain	Total	Strongly Disagree/ Disagree	Strongly Agree/ Agree
1	Before opioids	0%	0%	6%	24%	59%	12%	17	0%	83%
2	Before anti-epileptics	6%	53%	12%	12%	6%	12%	17	59%	18%
3	Before antidepressants without signs of major depressive disorder	6%	53%	12%	6%	12%	12%	17	59%	18%
4	Before acetaminophen (paracetamol) and NSAIDs	18%	65%	0%	6%	0%	12%	17	83%	6%
5	As a first-line therapy after non-pharmacotherapy options	24%	59%	0%	6%	0%	12%	17	83%	6%
