# Supplementary material for: Consensus recommendations on dosing and administration of medical cannabis to treat chronic pain: results of a modified Delphi process
Source: J Cannabis Res. 2021 Jul 2;3:22. doi: 10.1186/s42238-021-00073-1 (PMC8252988; doi:10.1186/s42238-021-00073-1)
Supplement: Supplementary file 4 — Additional file 4. First virtual meeting voting results. [file 42238_2021_73_MOESM4_ESM.pdf]

Consensus Virtual Voting Round 1 Results

Consensus Reached with ≥75%

n=20

The minimum age for use of THC in patients with chronic pain is:

1

#	Answer	Total	%
1	25 years of age	5	26.3%
2	21 years of age	4	21.1%
3	18 years of age	5	26.3%
4	No minimum age	5	26.3%
	Total	19	100.0%

The initiating THC:CBD ratio for patients with chronic pain requiring routine dosing and titration should be:

1

#	Question	Total	%
1	CBD predominant	13	65.0%
2	CBD predominant during the day and add THC predominant at bedtime	1	5.0%
3	Balanced THC:CBD	6	30.0%
4	THC predominant	0	0.0%
	Total	20	

The initiating CBD daily dose for routine dosing and titration is: (total amount in 24 hours)

1

#	Answer	Total	%
1	5 mg	1	5.0%
2	10 mg	15	75.0%
3	20 mg	1	5.0%
4	50 mg	3	15.0%
	Total	20	100.0%

The recommended CBD titration amount for routine dosing and titration is:

1

#	Answer	Total	%
1	5 mg	1	5.0%
2	10 mg	16	80.0%
3	20 mg	3	15.0%
	Total	20	100.0%

The recommended CBD up-titration frequency for routine dosing and adminstration is:

1

#	Answer	Total	%
1	Daily	0	0.0%
2	Every 2 to 3 days	17	85.0%
3	Once a week	3	15.0%
	Total	20	100.0%

The recommended maximum daily dose of CBD for routine dosing and adminstration is:

1

#	Answer	Total	%
1	100 mg	4	20.0%
2	300 mg	11	55.0%
3	No maximum daily dose	5	25.0%
	Total	20	100.0%

For routine dosing and titration, after initiating with a CBD predominant variety and target symptom relief has not been obtained, THC should be introduced:

1

#	Answer	Total	%
1	At bedtime once patient is taking >= 20mg of CBD	3	15.0%
2	At bedtime once patient is taking >= 40mg of CBD	15	75.0%
3	I do not recommend starting with CBD predominant before initiating THC	2	10.0%
	Total	20	100.0%

The recommended THC up-titration frequency for routine dosing and adminstration is:

1

#	Answer	Total	%
1	Every 2 to 3 days	14	70.0%
2	Once a week	6	30.0%
3	Every 2 weeks	0	0.0%
	Total	20	100.0%

The recommended maximum daily dose of THC for routine dosing and adminstration is:

1

#	Answer	Total	%
1	20 mg	0	0.0%
2	40 mg	14	70.0%
3	80 mg	5	25.0%
4	No maximum daily dose	1	5.0%

	Total	20	100.0%

If a patient is not achieving treatment goals with once daily THC at bedtime, clinicians can consider dosing THC up to:

1

#	Answer	Total	%
1	Twice daily	0	0.0%
2	Three times daily	12	60.0%
3	Four times daily	8	40.0%
	Total	20	100.0%

The initiating CBD daily dose for conservative dosing and titration is: (total amount in 24 hours):

1

#	Answer	Total	%
1	5 mg	5	29.4%
2	10 mg	8	47.1%
3	20 mg	4	23.5%
	Total	17	100.0%

The recommended starting dosing frequency for CBD is: (Daily dose divided into this frequency)

1

#	Answer	Total	%
1	Once daily	12	63.2%
2	Twice daly	7	36.8%
	Total	19	100.0%

The recommended CBD titration amount for conservative dosing and titration is:

1

#	Answer	Total	%
1	1 mg	0	0.0%
2	5 mg	7	38.9%
3	10 mg	9	50.0%
4	20 mg	1	5.6%
5	>20 mg	1	5.6%
	Total	18	100.0%

The recommended CBD up-titration frequency for conservative dosing and titration is:

1

#	Answer	Total	%
1	Daily	1	5.0%
2	Every 2 to 3 days	15	75.0%
3	Once a week	4	20.0%
4	Every 2 weeks	0	0.0%
	Total	20	100.0%

The recommended maximum daily dose of CBD for conservative dosing and titration is:

1

#	Answer	Total	%
1	50 mg	0	0.0%
2	100 mg	8	40.0%
3	300 mg	10	50.0%
4	No maximum daily dose	2	10.0%
	Total	20	100.0%

For conservative dosing, after initiating with a CBD predominant variety and target symptom relief has not been obtained, THC should be introduced:

1

#	Answer	Total	%
1	At bedtime once patient is taking >= 20mg of CBD	4	21.1%
2	At bedtime once patient is taking >= 40mg of CBD	15	78.9%
3	I do not recommend starting with CBD predominant before initiating THC	0	0.0%
	Total	19	100.0%

The recommended starting THC daily dose for patients with chronic pain requiring conservative dosing and titration: (amount for 24 hours)

1

#	Answer	Total	%
1	1 mg	17	94.4%
2	2.5 mg	1	5.6%
	Total	18	100.0%

The recommended THC titration amount is:

1

#	Answer	Total	%
1	1 mg	19	100.0%
2	2.5 mg	0	0.0%
	Total	19	100.0%

The recommended THC up-titration frequency for patients with chronic pain requiring conservative dosing and titration:

1

#	Answer	Total	%
1	Daily	0	0.0%
2	Every 2 to 3 days	9	45.0%
3	Once a week	10	50.0%
4	Every 2 weeks	1	5.0%

	Total	20	100.0%

The recommended maximum daily dose of THC for patients with chronic pain requiring conservative dosing and titration:

1	#	Answer	Total	%
	1	10 mg	1	5.0%
	2	20 mg	2	10.0%
	3	40 mg	15	75.0%
	4	80 mg	2	10.0%
	5	No maximum daily dose	0	0.0%
		Total	20	100.0%

If a patient is not achieving treatment goals with once daily THC at bedtime, clinicians can consider dosing THC up to:

1	#	Answer	Total	%
	1	Twice daily	6	30.0%
	2	Three times daily	11	55.0%
	3	Four times daily	3	15.0%
		Total	20	100.0%
