# Supplementary material for: Consensus recommendations on dosing and administration of medical cannabis to treat chronic pain: results of a modified Delphi process
Source: J Cannabis Res. 2021 Jul 2;3:22. doi: 10.1186/s42238-021-00073-1 (PMC8252988; doi:10.1186/s42238-021-00073-1)
Supplement: Supplementary file 5 — Additional file 5. Second virtual meeting results. [file 42238_2021_73_MOESM5_ESM.pdf]

Consensus Virtual Voting Round 2 Results

Consensus Reached with ≥75%

n=19

The initiating THC:CBD ratio for patients with chronic pain requiring routine dosing and titration should be:

1

#	Question	Total	%
1	CBD predominant	17	89.0%
2	CBD predominant during the day and add THC predominant at bedtime	1	5.0%
3	Balanced THC:CBD	1	5.0%
	Total	19	99.0%

The recommended maximum daily dose of THC for routine dosing and administration is:

1

#	Answer	Total	%
1	20 mg	0	0.0%
2	40 mg	18	94.7%
3	80 mg	1	5.3%
4	No maximum dose	0	0.0%
	Total	19	100.0%

The initiating CBD daily dose for conservative dosing and titration is: (total amount in 24 hours):

1

#	Answer	Total	%
1	5 mg	8	42.1%
2	10 mg	10	52.6%
3	20 mg	1	5.3%
	Total	19	100.0%

The recommended starting dosing frequency for conservative dosing for CBD is: (Daily dose divided into this frequency)

1

#	Answer	Total	%
1	Once daily	17	89.5%
2	Twice daly	2	10.5%
	Total	19	100.0%

The recommended THC up-titration frequency for patients with chronic pain requiring conservative dosing and titration:

1

#	Answer	Total	%
1	Every 2 to 3 days	0	0.0%
2	Once a week	16	84.2%
3	Every 5-10 days	3	15.8%
4	Every 2 weeks	0	0.0%
	Total	19	100.0%

The recommended maximum daily dose of THC for patients with chronic pain requiring rapid dosing and titration:

1

#	Answer	Total	%
1	40 mg	16	84.2%
2	60 mg	3	15.8%
3	80 mg	0	0.0%
	Total	19	100.0%

If a patient is not achieving treatment goals, clinicians can consider dosing:

1

#	Answer	Total	%
1	Twice daily	0	0.0%
2	Three times daily	9	60.0%
3	Four times daily	6	40.0%

	Total	15	100.0%

The starting dose of medical cannabis for inhaled use is:

1

#	Answer	Total	%
1	1 inhalation	15	83.3%
2	2 inhalations	2	11.1%
3	Abstain	1	5.6%
	Total	18	100.0%

The recommended daily dosing for inhaled cannabid is:

1

#	Answer	Total	%
1	Twice daily when required	0	0.0%
2	Four times daily when required	4	21.1%
3	Every 4 hours when required	14	73.7%
4	Abstain	1	5.3%
	Total	19	100.0%

The recommended up-titration amount for medical cannabis for inhaled use is:

1

#	Answer	Total	%
1	1 inhalation	16	84.2%
2	2 inhalations	2	10.5%
3	Abstain	1	5.3%
	Total	19	100.0%
