# Supplementary material for: Consensus recommendations on dosing and administration of medical cannabis to treat chronic pain: results of a modified Delphi process
Source: J Cannabis Res. 2021 Jul 2;3:22. doi: 10.1186/s42238-021-00073-1 (PMC8252988; doi:10.1186/s42238-021-00073-1)
Supplement: Supplementary file 6 — Additional file 6. Clinical experience and COI disclosures. [file 42238_2021_73_MOESM6_ESM.docx]

| **Author name** | **Academic affiliation** | **Clinical experience with medical cannabis** | **Disclosures of Conflicts of Interest** |
| --- | --- | --- | --- |
| Arun Bhaskar, MBBS MSc FRCA FFPMRCA FFICM FIPP EDPM | Head of Service & Consultant in Pain Medicine  Imperial College Healthcare NHS Trust, London  President, The British Pain Society  Hon. Secretary, Neuromodulation Society of UK & Ireland  Chapter President, International Association of Study of Pain  Councillor, European Pain Federation  Co-opted Council Member, Faculty of Pain Medicine  Section Chair UK, World Institute of Pain  Hon. Secretary, World Society of Pain Clinicians | Dr Bhaskar has been prescribing medical cannabis for eight years | Dr. Bhaskar has received speaker and consulting fees from Spectrum Therapeutics.  No family members have any relationship with Spectrum Therapeutics |
| Alan Bell, MD | Assistant Professor Department of Family and Community Medicine, University of Toronto | Dr. Bell has incorporated cannabis-based medicine in his clinical practice since 2001 when the MMAR (Marihuana Medical Access Regulations) were first enacted. | Dr. Bell has received speaker and consulting fees from Spectrum Therapeutics.  No family members have any relationship with Spectrum Therapeutics |
| Matthew Brown, MD (Res) FFPMRCA | The Royal Marsden Hospital, The Institute of Cancer Research | Prescriber  Principal investigator and co-investigator on UK’s largest studies in cannabis-based medicinal products in pain | Dr. Brown has received speaker and consulting fees from Spectrum Therapeutics.  No family members have any relationship with Spectrum Therapeutics |
| Hance Clarke, MD, PhD | Associate Professor, Department of Anesthesiology and Pain Medicine,  Toronto, Ontario  Canada | Dr. Clarke has been using cannabis-based medicine(s) in the chronic pain field since 2014. He is the co-founder of the UHN Centre for Cannabinoid Research. | Dr. Clarke declares Funding received from Medical Cannabis by Shoppers Drug Mart to perform a Real-World Evidence Trial in Canadian patients and an honorarium from Canopy Growth.  No family members have any relationship with Spectrum Therapeutics |
| Claude Cyr, MD | McGill University | Associate researcher Quebec Cannabis Registry. Approximately 200 active patients | Honorarium for CME speaker events, assisting and chairing advisory boards.  No family members have any relationship with Spectrum Therapeutics |
| Elon Eisenberg, MD | Institute of Pain Medicine, Rambam Health Care Campus, The Technion, Israel Institute of Technology, Haifa, Israel | Dr Eisenberg has been prescribing medical cannabis for 8 years | Dr. Eisenberg has received consulting fees from Spectrum Therapeutics.  No family members have any relationship with Spectrum Therapeutics |
| Ricardo Ferreira de Oliveira Silva, MD | Head of the spine and pain surgery department of Casa de Saúde São Jose – Rio de Janeiro - Brasil | Dr. Ricardo has been treating patients with medical cannabis for chronic pain since 2015 | Dr. Ricardo has received consulting fees from Spectrum Therapeutics.  No family members have any relationship with Spectrum Therapeutics |
| Eva Frohlich, MD | Specialist anaesthesiologist FFA (SA) (fellow of the faculty of anaesthesiologists of South Africa).  Wits medical center Johannesburg South Africa | Prescribing medical cannabis for 5 years. | Dr. Frohlich has received consulting fees from Spectrum Therapeutics.  No family members have any relationship with Spectrum Therapeutics |
| Peter Georgius, MD | Director of PainRehab, procedures.  Director of Rehabilitation Medicine, Selangor Nambour Private Hospital.  Director of Pain Management Programs at Selangor Nambour Private Hospital and Noosa Private Hospital.  Director of Sunshine Coast Clinical Research, Principal Investigator for studies in pain management, which include research into pharmacology and implantable devices. | PainRehab, specialises in complex pain conditions such as complex regional pain syndrome and complex neurological pain conditions. Cannabis therapy is used in the context of multidisciplinary pain management, pharmacology, physical therapies, psychology and advanced pain management procedures.  Dr Georgius has been treating patients with medical cannabis for 2 years | Dr Georgius received speaker and consulting fees from Spectrum Therapeutics.  No family member has a financial relationship with Spectrum Therapeutics  Dr Georgius receives fee from research with MedLab  Dr Georgius is a member of Medical Advisory Board of AusCann  Dr Georgius is a paid consultant for Abbott Medical and Boston Scientific |
| Malcolm Hogg, MD | Head of Pain Services, royal Melbourne Hospital. Clinical Associate Professor, University of Melbourne. | Dr Hogg has been using cannabis-based products in the management of chronic pain and spasticity since 2017. | Dr. Hogg has received speaker and consulting fees from Spectrum Therapeutics and Tilray Australia Pty Ltd.  No family members have any relationship with Spectrum Therapeutics |
| Tina Ingrid Horsted, MD | Anaesthesiologist, diploma in pain management | Dr Horsted has been prescribing medical cannabis for eight years | Dr. Horsted has received speaker and consulting fees from Spectrum Therapeutics.  No family members have any relationship with Spectrum Therapeutics |
| Caroline A. MacCallum, MD | Clinical Instructor Department of Medicine, Adjunct Professor Faculty Pharmaceutical. Sciences,  Associate Member Division of Palliative Care University of British Columbia | Dr. MacCallum has been using cannabis-based medicine(s) in the field of chronic pain and complex diseases since 2015. She has assessed and developed cannabinoid treatment plans for more than 5,000 patients.  She is the Medical Director of Greenleaf Medical Clinic, Canada’s first Medical Cannabis Clinic (2010). | Dr. MacCallum has received speaker and consulting fees from Spectrum Therapeutics  No family members have any relationship with Spectrum Therapeutics |
| Kirsten R. Müller-Vahl, MD | Professor of Psychiatry at the Department of Psychiatry, Socialpsychiatry and Psychotherapy at Hannover Medical School (MHH), Germany | Dr. Müller-Vahl has been using cannabis-based medicines primarily in the field of psychiatry and neurology since 2000. She is the first chairwoman of the German (ACM) and the international association for cannabinoid medicines (IACM). | Dr. Müller-Vahl has received consulting fees from Spectrum Therapeutics  No family members have any relationship with Spectrum Therapeutics |
| Colleen O’Connell, MD | Assistant Prof, Dalhousie University Faculty of Medicine, Physical Medicine and Rehabilitation | Clinician in neurorehabilitation, has provided education on pain and tone management and cannabinoids in neurologic conditions for 20 years nationally and internationally; founding member of the Canadian Consortium for the Investigation of Cannabinoids in 2000; has extensive 20-year experience with cannabis-based medicines in clinical practice. | Has served on advisory boards and provided education programs supported by honoraria from Canopy Growth, Tilray, Aurora. No family with Conflicts of Interest |
| Robert Sealey, MD | Cannabinoid Medicine Specialist, Victoria, British Columbia, Canada | Runs a medical cannabis clinic and has treated over two thousand patients with medical cannabis since 2001 | Dr. Sealey has received speaker and consulting fees from Spectrum Therapeutics.  No family members have any relationship with Spectrum Therapeutics. |
| Marc Seibolt, MD | Head of Algesiologikum Day Clinic for Pain Therapy  Munich, Bavaria, Germany | Dr. Marc Seibolt has been using cannabis-based medicine(s) in the chronic pain field since 2013. | Dr. Seibolt has received speaker and consulting fees from Spectrum Therapeutics.  No family members have any relationship with Spectrum Therapeutics. |
| Aaron Sihota, BScPharm, RPh | Clinical Instructor, Faculty of Pharmaceutical Sciences, University of British Columbia, Vancouver, BC, Canada | Clinician with direct patient care experience in a multidisciplinary referral only medical cannabis clinic for patients with pain, anxiety, and sleep issues. Managing patients since 2018.  Has educated at national and international symposia on the role of cannabinoids in the presence of opioids for managing pain control. | Has received speaker and consultancy honoraria from Canopy Growth. No family with Conflicts of Interest |
| Dustin Sulak, DO | Board member of the Society of Cannabis Clinicians | Dr. Sulak has been treating patients with cannabis-based medications in private practice since 2009. He is the medical director of Integr8 Health, a clinic that follows ~8,000 patients treated with cannabis in Maine, USA. | Dr. Sulak has participated in education and research events supported by honoraria from Spectrum Therapeutics. He is an equity owner and employee of Healer, paid member of an advisory board for COR Analytics, and paid member of an advisory board for Zelira Therapeutics. No family members have a relationship with Spectrum or other cannabis-related companies. |
| Antonio Vigano, MD | Associate Professor, Department of Oncology and  Co-lead biomedical axis, McGill Research Center for Cannabis, McGill University.  Attending physician, Supportive and Palliative Care Division and  Director, Cancer Rehabilitation Program and Medical Cannabis Program in Oncology,  Cedars Cancer Center, McGill University Health Center. | Dr. Vigano was appointed Expert on Medical Cannabis by the Court of Quebec, created the first world-wide post-doctoral research fellowship in medicinal cannabis and cancer supportive care within McGill’s Gerald Bronfman Department of Oncology, developed the first cannabis clinic within a quaternary oncology centre, and is Principal Investigator of the Quebec Cannabis Registry, a Canadian, prospective, province-wide, pharmaco-vigilance study on safety and effectiveness of medical cannabis, which recruited over 3000 patients with a follow up time period up to 4 years. | Has served on advisory boards and participated to education programs supported by honoraria from Canopy Growth and Tilray. Has been PI in clinical trials sponsored by TetraBiopharma which also provided funding for two post-doctoral research fellowships. Was research director at Sante’ Cannabis premiere Quebec based cannabis clinic, between January 2016 and March 2019. No family with Conflicts of Interest |
| Dwight E. Moulin, MD | Western University, London, Ontario | 25 years experience treating chronic pain with medical cannabis | Dr. Moulin has received speaker and consulting fees from Spectrum Therapeutics.  No family members have any relationship with Spectrum Therapeutics |
